# Influence of Disorder on the Electronic Properties and Magnetotransport of Ti_3_C_2_T_*x*_ Single-Flake Devices

**DOI:** 10.1021/acsaelm.5c01847

**Published:** 2026-01-08

**Authors:** Francesca Urban, Stefano Ippolito, Jane Frostad, José D. Gouveia, José R. B. Gomes, Paweł P. Michałowski, Steven J. May, Paolo Samorì, Yury Gogotsi

**Affiliations:** † A. J. Drexel Nanomaterials Institute, Department of Materials Science and Engineering, 6527 Drexel University, 3141 Chestnut St, Philadelphia, Pennsylvania 19104, United States; ‡ 27083 Université de Strasbourg & CNRS, ISIS & icFRC, 8 allee Gaspard Monge, 67000 Strasbourg, France; § CICECO - Aveiro Institute of Materials, Department of Chemistry, 56062 University of Aveiro, Campus Universitário de Santiago, 3810-193 Aveiro, Portugal; ∥ Łukasiewicz Research Network - Institute of Microelectronics and Photonics, al. Lotników 32/46, 02-668 Warszawa, Poland; ⊥ CICECO - Aveiro Institute of Materials, Department of Physics, University of Aveiro, Campus Universitário de Santiago, 3810-193 Aveiro, Portugal

**Keywords:** Two-dimensional materials, MXenes, charge transport, magnetoresistance, metal-to-disordered metal transition

## Abstract

The exploration of MXenes for electronic applications is a rapidly growing field in materials science. However, most research has focused on MXene films, with only a limited number of studies addressing the characterization of single-flake devices. In this work, we investigate the electronic and magnetotransport properties of Ti_3_C_2_T_*x*_ single-flake devices, exploring the influence of structural defectivity on their transport mechanisms. We show that negative magnetoresistance present at low temperatures in single flake samples arises from weak localization, which we analyze to extract the phase coherence length of single-layer and multi-layer flakes. The study of magnetoresistance for this metallic MXene shows that the material exhibits quantum transport phenomena when intrinsic electronic behavior dominates. Moreover, by increasing the defect density via thermal annealing in ultrahigh vacuum, we uncover and characterize the metal-to-disordered metal transition in Ti_3_C_2_T_*x*_, shedding light on new properties and enriching fundamental knowledge about MXenes.

## Graphical abstract

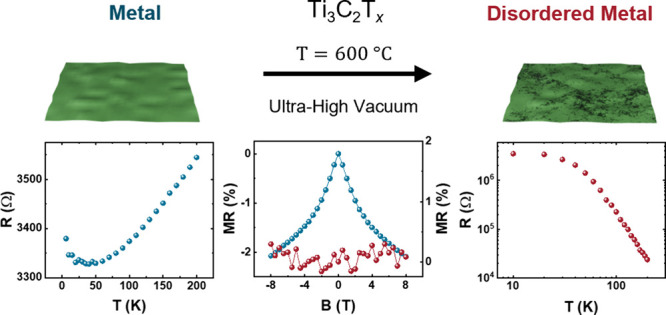

Due to the charge carrier confinement, two-dimensional (2D) materials represent ideal platforms to investigate quantum phenomena in low-dimensional regimes.^1,2^ MXenes are 2D transition metal carbides, nitrides and carbonitrides having the general formula M_*n*+1_X_*n*_T_*x*_, where M stands for an early transition metal, X is carbon and/or nitrogen, T_*x*_ refers to surface terminations (generally −O, −OH and −F groups), while *n* ranges from 1 to 4.^3^ MXenes have attracted ever-growing interest because of their metallic conductivity, high processability in environmentally friendly solvents (including water), as well as tunable density of states and work function.^4^ However, despite the great potential in a wide range of technologies,^5–7^ their fundamental intrinsic electronic properties, including the dependence on structural defectivity and ability to display quantum transport phenomena, have been investigated to a much lesser extent compared to graphene and other 2D materials. In particular, the elucidation of charge transport and quantum phenomena in MXenes is hindered by their compositional and structural complexity, which is enhanced by the presence of defects and/or adsorbates depending on the chemical routes utilized during the synthesis protocols. Thus far, most transport studies were conducted on free-standing or thin films, strongly affected by several extrinsic factors including inter-flake junction resistance, the presence of inter- and/or intra-layer contaminants, such as intercalated ions from the delamination steps, mixture of surface terminations, variable interlayer spacings (i.e., structural voids), and occurrence of oxidation. Often, such characteristics can prevail over the intrinsic intra-flake transport mechanisms and lead to a significant increase in the sample resistivity, with inconsistent results recorded from temperature-dependent investigations.^8–10^ Thus far, experiments carried out on single-flake devices demonstrated how synthesis conditions and oxidation level influence the temperature-dependent electrical properties.^11,12^ However, a significant gap in the understanding of fundamental properties and their relationship with the crystal structure defectivity still remains. In this Letter, we report on the charge- and magnetotransport of Ti_3_C_2_T_*x*_ single-flake devices, emphasizing the effects of increasing structural defectivity and disorder through ultrahigh vacuum thermal annealing steps. As a result of such treatments, we demonstrate that a change in the temperature-dependent electronic behavior takes place at the single-flake level, even without oxidation to TiO_2_, and this can be ascribed to increasing crystal disorder.

Ti_3_C_2_T_*x*_ MXene was synthesized via wet-chemical etching and lithium-ion intercalation following previously reported protocols^13^ (Supporting Information Section 1). For the device fabrication, Ti_3_C_2_T_*x*_ flakes were deposited via dip coating onto SiO_2_/Si substrates and contacted in a two-probe geometry using photolithography (Supporting Information Section 2).

The schematic and optical micrograph of a typical device are displayed in Figure 1(a-b). Atomic force microscopy (AFM) and Raman spectroscopy were used to determine the flake thickness and quality, respectively (Supporting Information Section 3–4). The charge transport properties were investigated for flakes of different thicknesses, ranging from single- (1L) to multi-layer (ML). The resistance *vs* temperature curves, from 5 to 300 K, recorded for two representative 1L and ML samples, are displayedin Figure 2(a). Both devices exhibit typical metallic behavior (*viz*, *dR*/d*T* > 0) and *R* ∝ *T* ^3^ (Bloch-Gruneisen model) with a resistance upturn at *T* < 80 K. The inset of Figure 2(a) compares the mean value of the temperature of minimum resistance (*T*_*min*_) for the two systems under investigation (Supporting Information Section 6–7). The resistance upturn has already been reported for Ti_3_C_2_T_*x*_ thin films and it was tentatively ascribed to weak localization (WL), due to structural and morphological disorder, partial oxidation, inter-flake transport and change in the transport dimensionality.^8,11,14^

**Figure 1:**
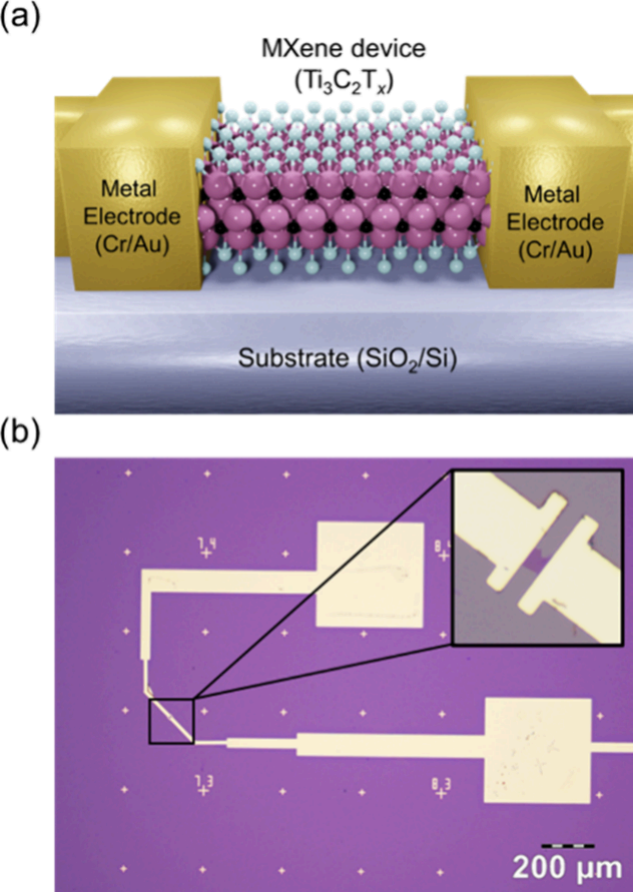
(a) Schematic and (b) optical micrograph of a single-flake Ti_3_C_2_T_*x*_ device.

**Figure 2:**
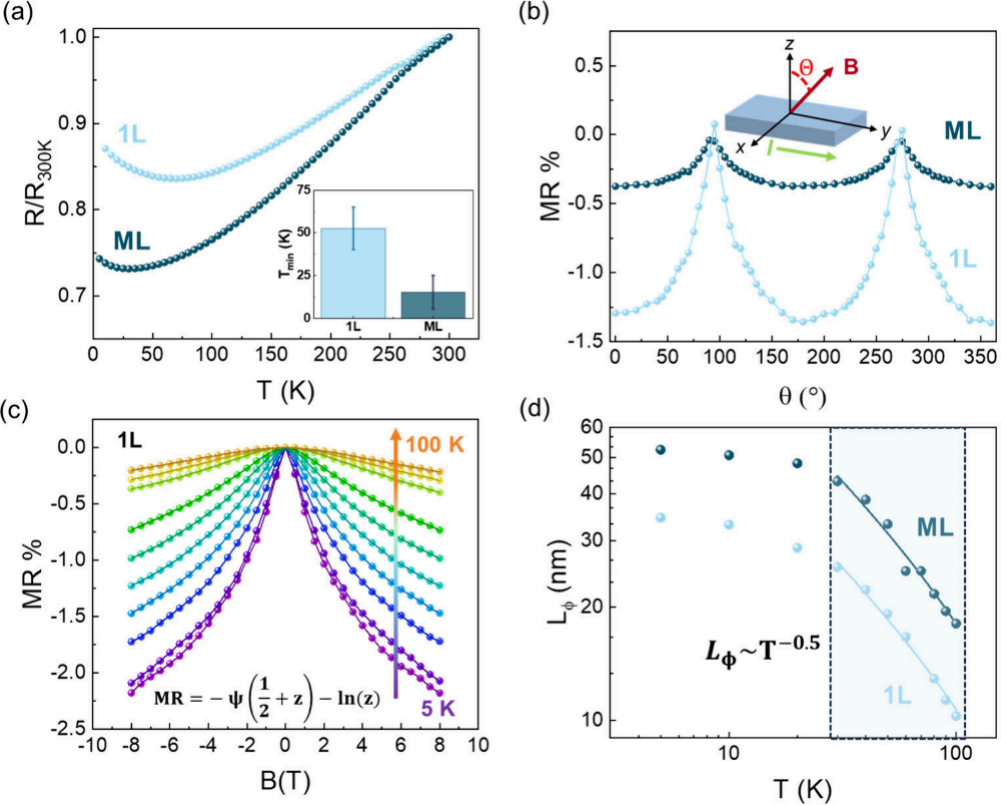
(a) Normalized resistance as a function of temperature for two representative 1L and ML Ti_3_C_2_T_*x*_ devices. The data were recorded at constant current of 10 μA. The inset highlights the difference in *T*_*min*_ between flakes of different thickness. (b) Angle-dependent MR (*T* = 10 K, *B* = 8 T) for two representative 1L and ML devices. The inset shows the geometric relationship between the field (*B*), the angle (θ), and current (*I*) of the sample. (c) Typical temperature evolution of field-dependent MR for a 1L device. The solid lines at each temperature describe the fitting using the Hikami-Larkin-Nagaoka model, reported in eq 1. (d) Phase coherence length as a function of temperature for 1L and ML devices, where the solid line displays a *T* ^–0.5^ trend at temperatures above 20 K.

From the temperature-dependent measurements on single flakes, we can unambiguously state that the resistance upturn and WL are intrinsic features of the material, as well as correlate the observed trend for the *T*_*min*_ and magnitude of the resistance upturn to the flake thickness, with MLs showing lower *T*_*min*_ and smaller upturn than 1Ls (Figure S4). This might be due to the increasing structural disorder and defect density in thinner flakes,^11^ which leads to a stronger localization of the charge carriers and, thus, higher *T*_*min*_ values.^15,16^ The statistical analysis on the resistance upturn and flake-to-flake variance is reported in Supporting Information Section 9. To confirm our hypothesis and rule out other possible mechanisms taking place at low temperature (e.g., Kondo effect), we performed magnetoresistance (MR) measurements. Despite the paramagnetic nature of Ti_3_C_2_T_*x*_, negative MR is observed with a strong angular dependence on the applied magnetic field (*B*), as displayed in Figure 2(b). The angle of the magnetic field (θ) is defined as 90° and 270° (0° and 180°) when the magnetic field is out-of-the-plane (in-plane); the scheme of the angular-dependent MR measurements is depicted in the inset of Figure 2(b). This behavior is consistent with previous observations of negative MR and field-induced increase of source-drain current reported at low temperature in Ti_3_C_2_T_*x*_ films^8,14^

The solid lines in Figure 2(c) represent the fitting curves according to the Hikami-Larkin-Nagaoka (HLN) model^16^ (Supporting Information Section 12) for diffusive metals with high spin–orbit coupling (*τ*_*ϕ*_ ≫ *τ*_*SO*_):

1 $$\mathrm{MR}=-\alpha \frac{e^{2}}{\pi h}\left[\psi \left(\frac{1}{2}+\frac{B_{\phi }}{\left|B\right|}\right)+\ln\frac{B_{\phi }}{\left|B\right|}\right]$$

where *τ*_*ϕ*_ and *τ*_*SO*_ are the phase coherence and spin–orbit scattering, respectively, ψ is the digamma function, *B* the applied field, *B*_*ϕ*_ the phase breaking field. *B*_*ϕ*_ and α represent the fitting parameters that determine the shape of the curve. From *B*_*ϕ*_ we can estimate the phase coherence length:

2 $$L_{\phi }=\sqrt{\frac{\hbar }{4eB_{\phi }}}$$

which describes the distance that electrons travel before the phase coherence is destroyed. Using eq 1 and 2, we extrapolated two representative fits of *L*_*ϕ*_ *vs* temperature, displayed in Figure 2(d), for two representative 1L and ML devices. The phase coherence lengths show a *T* ^–0.5^ dependence, before saturation is reached at a low-temperature regime (below 20 K), a behavior widely reported for other 2D systems with similar *L*_*ϕ*_ in the order of the tens of nm.^17–19^ This is often ascribed to surface effects, presence of interfaces and substrate interaction, along with additional contributions coming from the spin–orbit coupling,^15,17,20^ mostly related to the inherent lattice disorder and defectivity.

Moreover, the dependence of *T*_*min*_ and *L*_*ϕ*_ on the thickness, as well as the correlation between MR and *T*_*min*_, confirm that the negative MR arises from the localization mechanism induced by local defects and disorder, less pronounced in thicker ML samples. A recent work on Ti_3_C_2_T_*x*_ films reported a decrease in *L*_*ϕ*_ after increasing ion irradiation,^14^ being consistent with our claim that structural defectivity plays a key role and influences the overall electronic transport.

To further explore the relationship between electrical properties and structural defectivity, we performed thermal annealing treatments in ultrahigh vacuum (10^–8^ mbar) at three different temperatures, namely 200, 400, and 600 °C (Supporting Information Section 5). The highest annealing temperature (i.e., 600 °C) promotes the formation and propagation of structural defects, as widely reported in the literature.^21–23^ While minimal changes in the flake resistivity were recorded for the samples treated at 200 and 400 °C (Figure S5 and Figure S6), a drastic change in the temperature-dependence behavior was observed after 600 °C annealing, regardless of the flake thickness (Figure S7). The major change in the resistance *vs* temperature data set for a 1L device before and after 600 °C annealing is portrayed in Figure 3(a). It is worth mentioning that the annealed flakes were directly measured in vacuum, thus they were not exposed to air after the annealing treatment in order to avoid physisorption and/or intercalation of water on the flake and between the layers. The data in Figure 3(a) are displayed on a *log–log* scale to emphasize the trend of the annealed 1L sample (red curve), revealing a decrease in resistance when increasing the temperature. In metals, the negative temperature derivative of the resistance (d*R*/d*T*) can be an indication of two types of transition: (i) metal-to-insulator and (ii) metal-to-disordered metal, whose differences can only be spotted when plots are reported in *log–log* scale. In the disordered metal regime, the resistance saturates at low temperature, and such a behavior correlates to the electron–electron interaction and breakdown of the Matthiessen’s rule caused by structural defectivity of the system.^24^ Disordered or glassy metals are known to exhibit electronic features determined by the overall system defectivity, with a temperature-dependent behavior similar to the semiconducting regime and characterized by a negative slope of the resistanc*e vs* temperature curve.^24,25^ The observed *dR*/d*T* < 0 trend in Figure 3(a) has been already reported in the MXene literature for Ti_3_C_2_T_*x*_ films^8–10^ and other MXenes and ascribed to inter-flake hopping.^26,27^ However, our demonstration of such behavior in single-flake devices definitely rules out the predominance of the inter-flake charge transport mechanism, attributing the *dR*/d*T* < 0 to a change in the intra-flake charge transport. The saturation of the resistance at low temperature, in the *log–log* scale plot, attribute such a behavior to the MXene transition to a disordered-metal state.^25,28^

**Figure 3:**
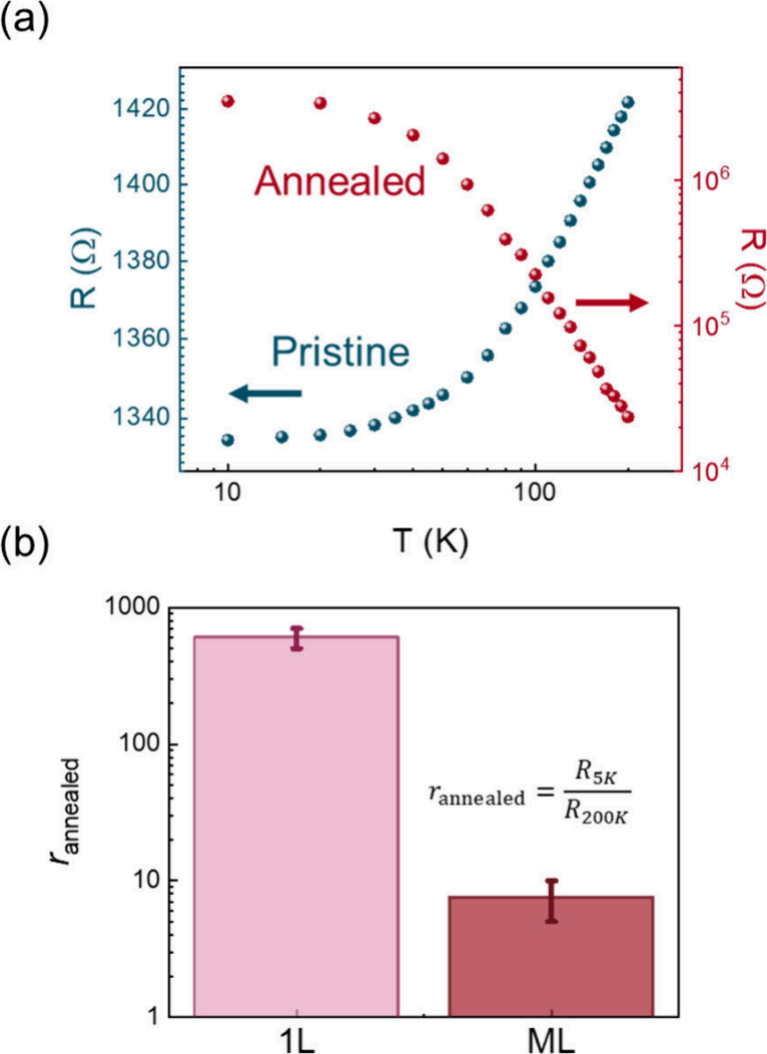
(a) Resistance *vs* temperature curves before and after 600 °C annealing for a typical 1L device. (b) Average *r*_annealed_ values for 1L and ML devices.

The ratio between the resistance at low and high temperature shown in Figure 3(b) can be considered as an indication of the degree of disorder within the system, which shows a dependence on the flake thickness, in agreement with the results reported above for WL and MR analysis. In this regard, a difference of almost 2 orders of magnitude is observed between 1L and ML devices. Thus, the flake thickness influences the final structural defectivity following the 600 °C annealing, with 1Ls being on average more defective than MLs upon treatment. Moreover, the correlation between the absolute value of the residual resistivity and the *dR*/d*T* sign allows for an indicative evaluation of the resistivity range for which the metal-to-disordered metal transition happens, according to the Mooij criterion (Figure S13, Supporting Information Section 10).

From a theoretical standpoint, we report the calculation of the Ti_3_C_2_T_*x*_ band structure via density functional theory (Supporting Information Section 15), varying the density and type of defects, as well as the nature of the surface terminations. Our calculations show that the metallic nature of Ti_3_C_2_T_*x*_ is always retained even at high Ti vacancy densities (Figure S22).

In addition, to monitor the changes in the material structure with atomic resolution, we performed secondary ion mass spectrometry (SIMS) investigations, transmission electron microscopy (TEM) analysis and electron diffraction on pristine and 600 °C-annealed samples (Supporting Information Sections 16–17).^29,30^ From SIMS analysis, pristine samples exhibit a typical depth profile of a single-layer flake, enabling a precise determination of the chemical composition of each layer [Figure 4(a)]. In the X layers, we found carbon and oxygen contents of 86.5% and 13.5%, respectively. On the other end, 600 °C-annealed samples displayed a markedly different scenario [Figure 4(d)]. The termination layers are predominantly composed of oxygen moieties, whereas we found that fluorine and chlorine terminations are completely removed during the annealing at 600 °C, with the average carbon and oxygen concentrations decreasing to 76.5 and 9.4 at%, respectively. Contrary to the pristine sample, the carbon and oxygen content in the annealed samples does not sum up to 100%, indicating the formation of about 14 at% of vacancies within such layers. Moreover, all samples show a distinct noise, with an amplitude of fluctuations about 1 order of magnitude higher than the pristine flakes, pointing to an increase of the structural defectivity, also in the M lattice (e.g., Ti misplacement), upon annealing. A similar behavior was observed for a sample where numerous structural defects were induced by ion implantation (Supporting Information Section 14). Therefore, the higher noise level can be unambiguously ascribed to increased defectivity in the lattice induced by thermal annealing. Similar considerations apply to the TEM analysis. The pristine sample shows a uniform surface (Figure 4(b)), while we observe the appearance of a “patchy” texture after the annealing at 600 °C (Figure 4(e)). We can attribute such a contrast difference to the formation of locally thicker and thinner areas due to the Ti and C dislocation.^23^ Upon thermal treatment, the electron diffraction pattern changes from the typical hexagonal pattern for pristine Ti_3_C_2_T_*x*_ (Figure 4(c)) to a more complex pattern with lower symmetry and additional diffraction spots (Figure 4(f)), with analogous distance values for pristine and annealed samples (Figure S25). The lack of typical diffraction spots for TiO_2_, both rutile and anatase phases, further corroborates the lack of oxidation in our material (Supporting Information Section 17). Our investigation of Ti_3_C_2_T_*x*_ single-flake devices offers new insights into the role of defects and disorder on transport properties. The charge- and magnetotransport analysis confirmed that the resistance upturn at low-temperature is due to weak localization. We also observed a negative MR, characterized by field and angle dependence, in agreement with the WL model, whose magnitude correlates with the onset temperature for the resistance upturn. By fitting the MR data, we obtained phase coherence lengths in the range from 30 nm for 1L devices to 60 nm for ML devices, along with *T* ^–0.5^ behavior for both systems. This proves that metallic MXenes can exhibit quantum transport phenomena when probed in the single-flake limit, where intrinsic electronic behavior dominates. Moreover, through thermal annealing steps and related changes in the structural defectivity, we corroborated the hypothesis that X-site as well as Ti vacancies^22^ strongly impact the MXene electronic properties. In particular, from temperature-dependent electrical analysis, a change in the slope of *dR/dT* was observed after 600 °C vacuum annealing. Our study on the electronic properties of MXenes expands the fundamental knowledge and offers new guidelines to further explore low-temperature transport, critical behaviors, and quantum phenomena in such materials.

**Figure 4:**
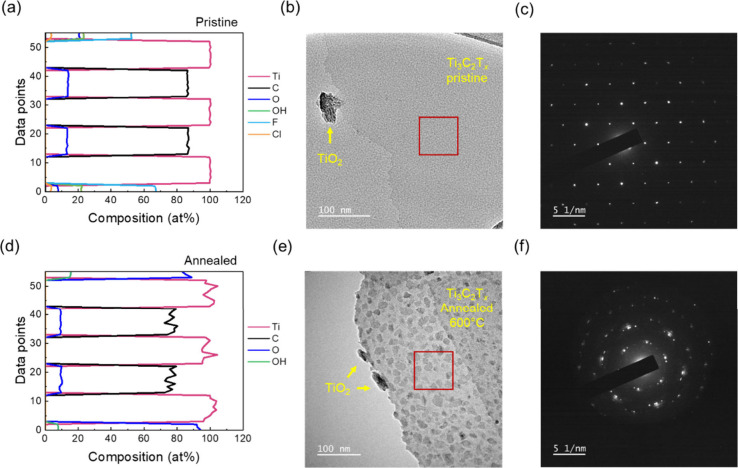
Secondary ion mass spectrometry analysis, TEM images, and electron diffraction patterns corresponding to the red square, performed on 1L (a-c) pristine and (d-f) 600 °C-annealed devices.

## Supplementary Material



## Notes

Additional materials, methods and experimental details (PDF)
