# Supplementary material for: Influence of Disorder on the Electronic Properties and Magnetotransport of Ti3C2T x Single-Flake Devices
Source: ACS Appl Electron Mater. 2026 Jan 8;8(2):732–7. doi: 10.1021/acsaelm.5c01847 (PMC12854750; doi:10.1021/acsaelm.5c01847)
Supplement: Supplementary file 1 [file el5c01847_si_001.pdf]

Supporting Information

Influence of disorder on the electronic properties and magnetotransport of $Ti_3C_2T_x$ single-flake devices

Francesca Urban [a,b], Stefano Ippolito [a], Jane Frostad [a], José D. Gouveia [c], José R. B. Gomes [d], Paweł P. Michałowski [e], Steven J. May [a], Paolo Samorì [b], Yury Gogotsi [a*]*

[a] A. J. Drexel Nanomaterials Institute and Department of Materials Science and Engineering, Drexel University, 3141 Chestnut St, Philadelphia, PA 19104, USA

[b] University of Strasbourg & CNRS, ISIS & icFRC, 8 allee Gaspard Monge, 67000 Strasbourg, France

[c] CICECO – Aveiro Institute of Materials, Department of Physics, University of Aveiro, Campus Universitário de Santiago, 3810-193 Aveiro, Portugal

[d] CICECO – Aveiro Institute of Materials, Department of Chemistry, University of Aveiro, Campus Universitário de Santiago, 3810-193, Aveiro, Portugal

[e] Łukasiewicz Research Network - Institute of Microelectronics and Photonics, al. Lotników 32/46, 02-668 Warszawa, Poland

Corresponding authors: gogotsi@drexel.edu; samori@unistra.fr

Table of contents

1. Material synthesis ____ 2
2. Device fabrication ____ 2
3. Atomic force microscopy (AFM) ____ 3
4. Raman spectroscopy ____ 3
5. Thermal treatment ____ 4
6. Temperature-dependent measurements and charge transport analysis ____ 4
7. R *vs.* T fittings : Bloch-Gruneisen and Weak localization ____ 8
8. W-function method and α evaluation ____ 9
9. Flake-to-flake variance ____ 10
10. Mooij criterion ____ 11
11. I-V curves before and after temperature annealing ____ 12
12. Magnetoresistance analysis ____ 13
13. Magnetoresistance measurements after thermal treatment ____ 15
14. Secondary ion mass spectrometry ____ 16
15. Density functional theory calculations ____ 17
16. Scanning electron microscopy and energy dispersive spectroscopy ____ 21

17. Transmission electron microscopy 21
18. References 25

1. Material synthesis

$Ti_3C_2T_x$ was produced by selective wet-chemical etching following a previously described protocol[1] schematically summarized in Figure S1. One gram of commercial Ti_3AlC_2 MAX powder (Carbon-Ukraine) was slowly added to 20 mL of etchant and stirred at 300 rpm at 35 °C for 24 h. The etchant was a 6:3:1 mixture (by volume) of 12 M HCl, deionized (DI) water, and 50 wt.% HF (Acros Organics). Multilayered $Ti_3C_2T_x$ MXene was then intercalated with LiCl (1 g of LiCl per gram of MAX phase) dissolved in 50 mL of DI water and stirred at 300 rpm at 35 °C for 24 h. The resulting solution was washed with DI water and centrifuged at 3500 rpm for 5 min. The supernatant was discarded, and the delaminated MXene was redispersed by manual shaking. The washing procedure was repeated until pH > 6. Finally, the colloidal solution was centrifuged at 3500 rpm for 60 min, and the supernatant containing delaminated single $Ti_3C_2T_x$ flakes was collected.

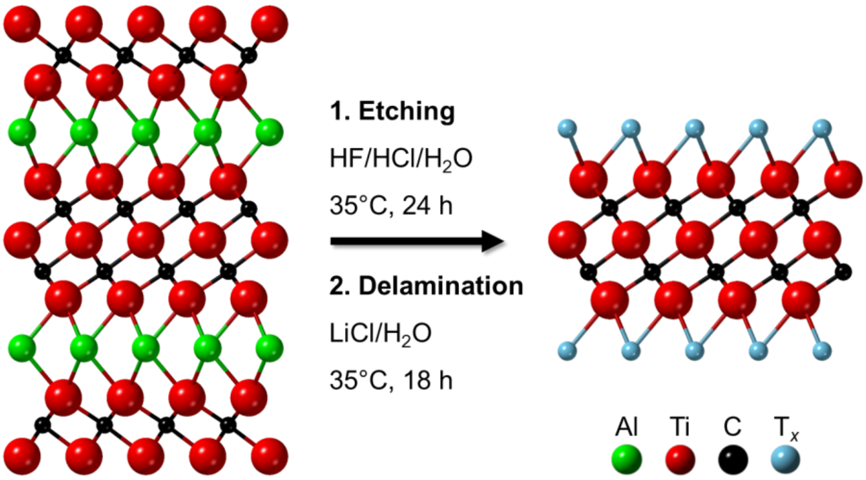

Figure S1. Schematic displaying the synthesis of $Ti_3C_2T_x$ single flakes via wet-chemical etching protocol.

2. Device fabrication

SiO_2/p^{++} Si substrates (10 × 10 mm^2, Fraunhofer IPMS) were used, having thermally grown SiO_2 (300 nm thick). Before use, the substrates were cleaned by ultrasonication in acetone and 2-propanol (10 min each) and dried under nitrogen flow afterward. $Ti_3C_2T_x$ flakes were deposited by dip coating (15 min) upon substrate surface activation via oxygen plasma (5 min). Towards this end, an aqueous dispersion of $Ti_3C_2T_x$ with a concentration equal to 0.05 mg/mL was used. Single flakes were identified via optical microscopy, and their thickness was measured using atomic force microscopy. Then, the devices were fabricated by photolithography (AZ1505 photoresist and MIF326 developer, MicroChemicals) using Microtech LW405B laser writer. Metal electrodes were made of 5 nm of chromium and 40 nm of gold, thermally evaporated with Plassys MEB 300,

followed by lift-off in warm (40 °C) acetone for 90 min. Finally, the devices were rinsed with acetone and 2-propanol to remove photoresist and gold residues. The device channel lengths and width vary from sample to sample, depending on the flake size.

3. Atomic force microscopy (AFM)

Topographical AFM images, as in Figure S2, were recorded with Bruker Dimension Icon microscope under ambient conditions, operating in tapping mode and using TESPA-V2 tips with a spring constant, $k = 42$ N/m. Images were captured at a scan rate of 1 Hz with 512/1024 lines per image.

We calculated the number of layers for the $Ti_3C_2T_x$ flake via AFM analysis. The typical flake morphology and thickness for a ML flake are displayed in Figure S2. Our ML samples range from 6 to 15 nm, meaning ca. 4 to 10 MXene layers (we considered for 1L $Ti_3C_2T_x$ flake an average value of 1.5 nm).

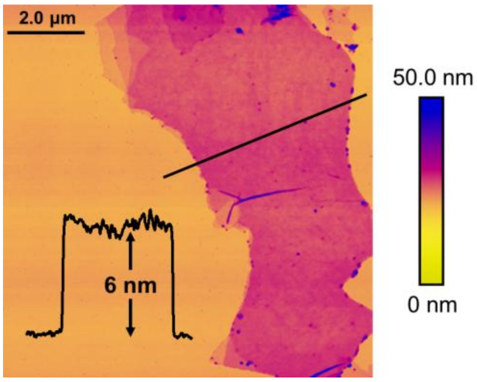

Figure S2. Atomic force microscopy image of a few-layer MXene flake on SiO_2/Si substrate (inset: height profile recorded along the black line).

4. Raman spectroscopy

The Raman spectra were taken at a 785 nm laser wavelength. The $Ti_3C_2T_x$ deconvoluted Raman spectrum, displayed in Figure S3, is dived into 3 regions: the flake region, which corresponds to a group vibration of carbon, two titanium layers, and surface groups, the T_x region, which represents vibrations of the surface groups, and the carbon region, where both in-plane and out-of-plane vibrations of carbon atoms are located, as described in ref [2].

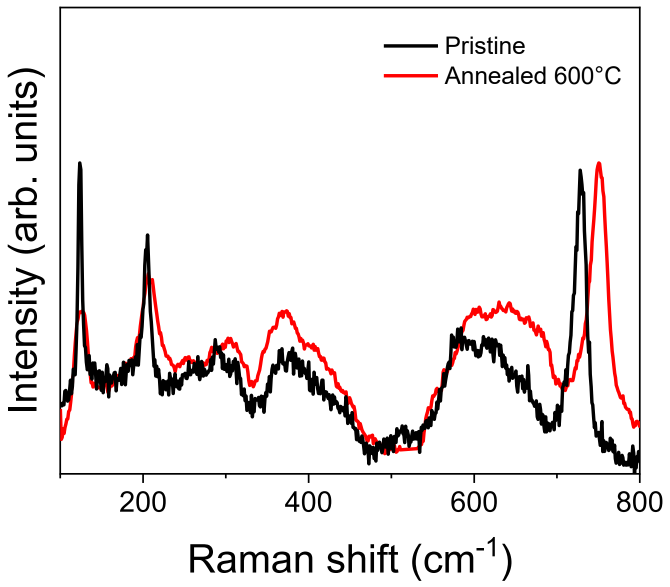

Figure S3. Raman spectrum of a few-layer $Ti_3C_2T_x$ flake before and after 600°C annealing.

The broadening and shift of the Raman peaks after 600°C annealing can indicate: (i) the formation of defects in the lattice and an increase in the crystal disorder (as reported for other 2D materials for which Raman studies of defectivity have been extensively conducted[3,4]); (ii) a change in the surface terminations[5].

5. Thermal treatment

After the first round of electrical measurements, the devices were annealed in an ultra-high vacuum (~ 10^{-8} mbar) at three different temperatures (200 °C, 400 °C, and 600 °C) in a Plassys evaporator located inside a nitrogen-filled glovebox. To avoid contamination, physisorption, and water intercalation after the annealing procedure, the samples were sealed in the glovebox and carried to a Janis Cryogenic Probe Station (PS) through a customized vacuum mini-chamber. The mini-chamber was loaded and opened inside the PS once in vacuum.

6. Temperature-dependent measurements and charge transport analysis

The electrical devices were measured in a two-probe configuration in a customized Cryogenic Janis PS, connected to a semiconductor parameter analyzer (Keithley 4200A SCS). The chamber pressure was kept at ~10^{-7} mbar. A Sumitomo RDK-408D2 cryogen-free closed cycle is thermally anchored to the copper sample holder, and the temperature measurements were performed within the temperature range of 200 to 5 K.

For comparison, we conducted the same experiments in a Lakeshore Physical Property Measurement System (PPMS) within the 5-300 K range, bonding the device pads with silver paste. In the cryogenic PS, the temperature measurements were carried out by performing the current-voltage (I-V) characterization, sweeping the voltage from -100 to 100 mV at fixed temperatures. On the contrary, in the PPMS, the temperature measurements were performed at a fixed current (10 μA). We characterized the devices as prepared (pristine) and after each temperature annealing. To ensure the comparison between the devices and the different experimental conditions and statistically monitor the changes, we measured the pristine state before each annealing. The resistance (R) of pristine $Ti_3C_2T_x$ flakes of different thicknesses at high temperatures behaves

like a conventional metal, while below ~50 K, the resistance exhibits an upturn and increases with decreasing temperature, as reported below (Figure S4).

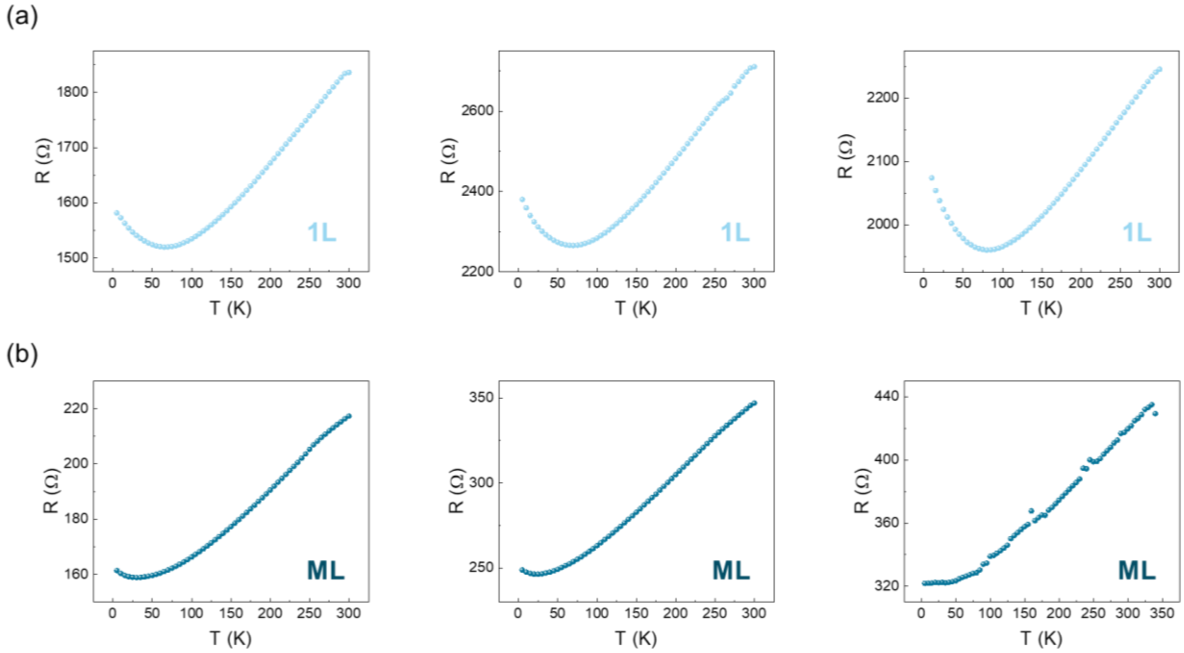

Figure S4. Resistance vs. temperature curves in three typical (a) monolayer (1L) and (b) multilayer (ML) devices.

The difference in the resistance upturn correlates to the flake-to-flake variability, arising from the etching and the material dispersion.

After the initial characterization, the devices were subjected to UHV (10^{-8} mbar in an evaporator chamber located in an N_2 glovebox) annealing at 200 °C, 400 °C, and 600 °C to remove the intercalated water, -OH, and -F/-Cl surface terminations, respectively, and propagate/induce structural defects (*e.g.*, vacancies) in the crystal lattice. The removal of water (200 °C) and -OH groups (400 °C) is observed not to influence the metallic behavior and the charge transport properties of the material, as reported in Figure S5 and S6, respectively, where a comparison of the curve before and after the two annealing processes is displayed for other two representative sets of samples (1L and ML).

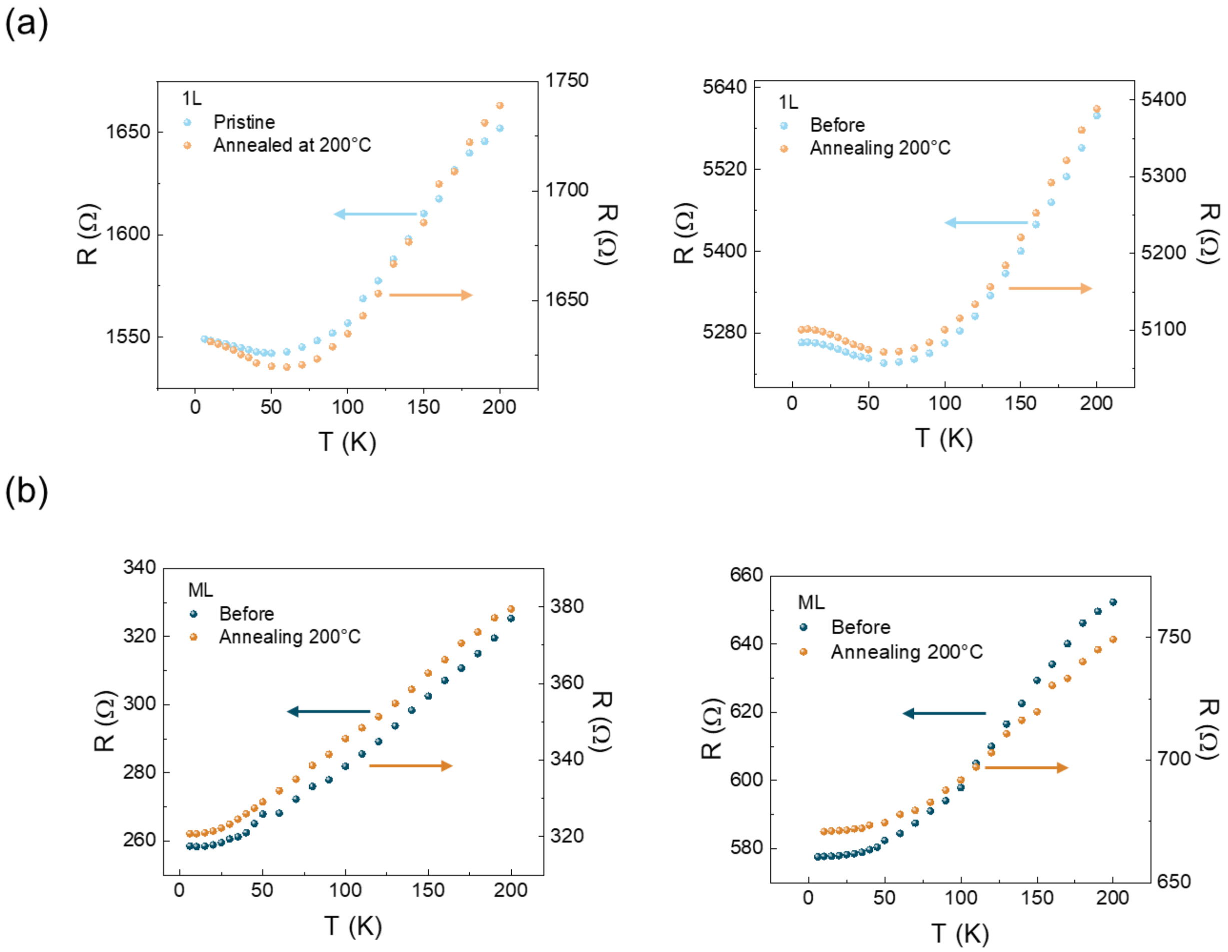

Figure S5. Resistance vs. temperature in (a) monolayer and (b) multilayer devices after 200 °C annealing.

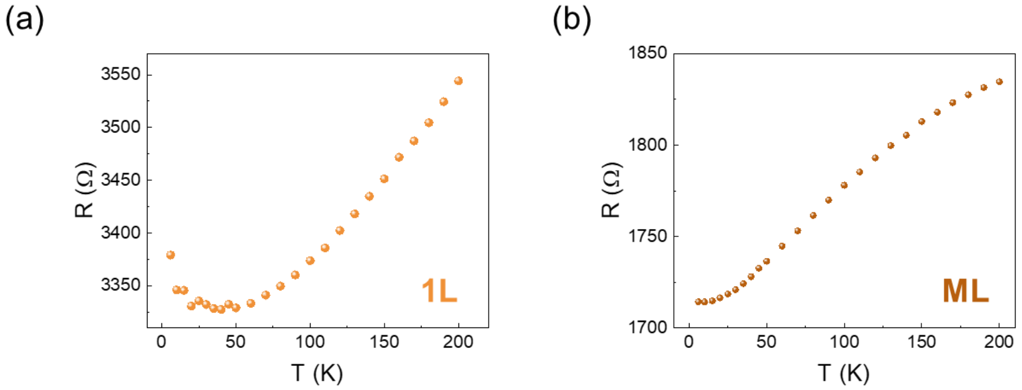

Figure S6. Resistance vs. temperature in (a) monolayer (1L) and (b) multilayer (ML) devices after 400 °C annealing.

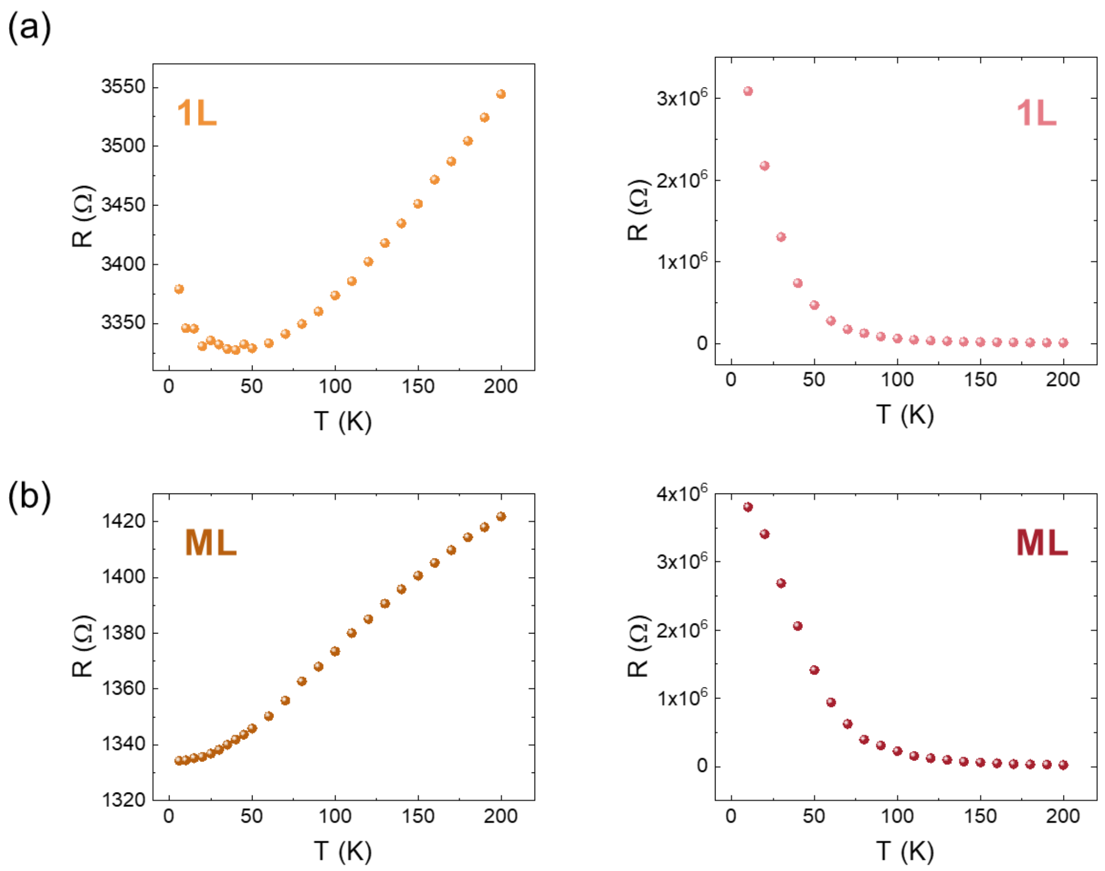

Figure S7. Resistance vs. temperature in (a) monolayer and (b) multi-layer devices before (left panels) and after 600 °C annealing (right panels). They correspond to the samples reported in Figure S6.

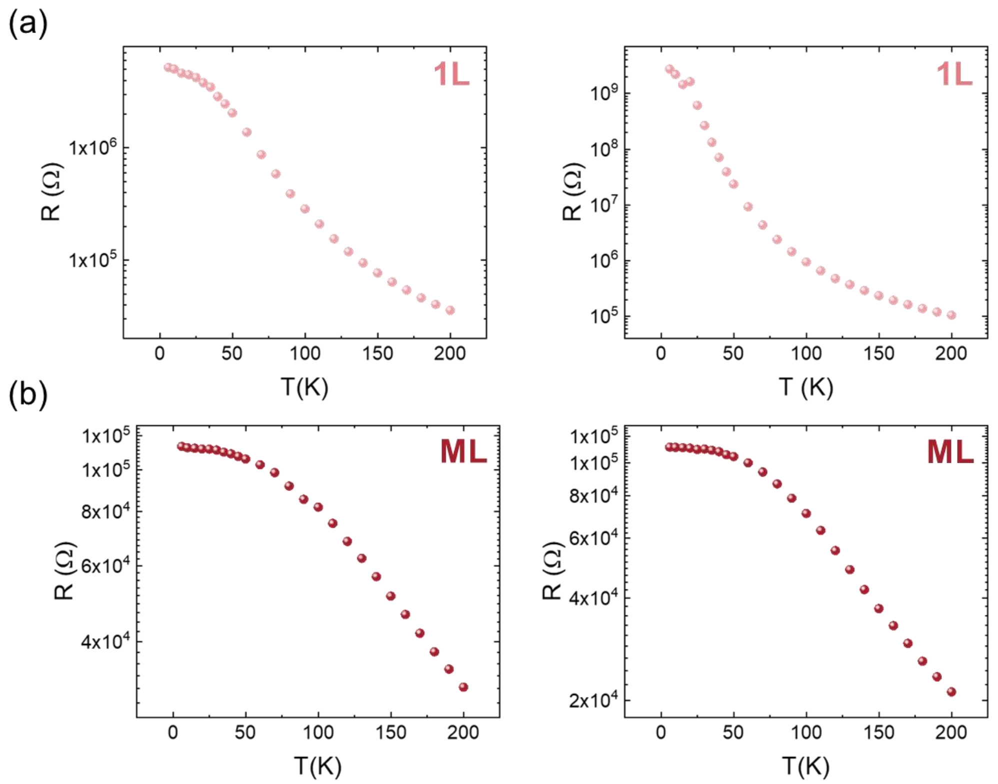

Figure S8. Resistance vs. temperature in (a) monolayer and (b) multi-layer devices after 600 °C annealing.

After high-temperature annealing (600 °C), a change in the slope of dρ/dT is often observed in the resistance vs. temperature behavior. An overall increase of the material resistance is reported, probably due to the increase of the structural defectivity, as well as a negative trend of the resistance *vs.* temperature curves ($dR/dT < 0$). We want to underline that this transition appears to be independent of the flake thickness, as reported in Figures S7 and S8, and the saturation of the curves at low temperature is indicative of a metal to disordered metal transition, as reported in Figure 3(a) of ref.[6]. The dependence of this transition on the flake-to-flake variance is reported in Section 9.

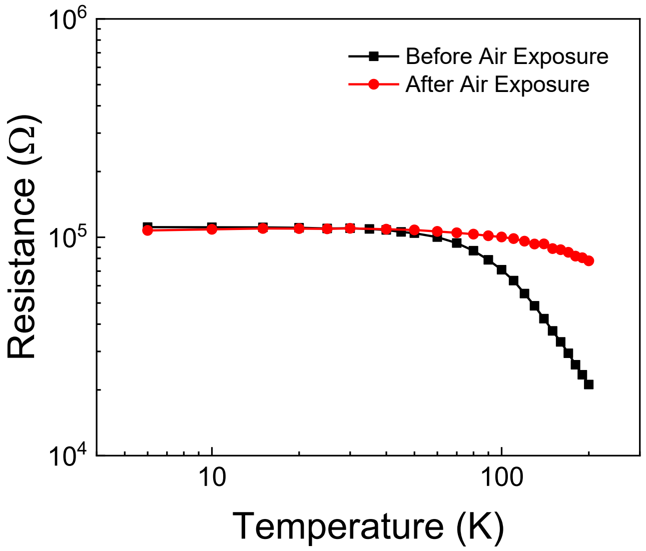

Figure S9. Resistance vs. temperature in multi-layer devices treated at 600 °C before and after air exposure.

To consider the effect of water and/or adsorbates on the electrical properties, we performed a comparison of *R vs T* for ML $Ti_3C_2T_x$ single-flake devices by measuring their performance right after the thermal annealing and upon exposure to air overnight. As displayed in Figure S9, only a minimal increase in resistance is recorded (a factor of 2-3), still showing a similar trend and saturation at cryogenic temperatures, confirming that the effect of the annealing on the electrical behavior is permanent and not influenced by water/adsorbates.

7. *R vs. T* fittings: Bloch-Gruneisen and Weak localization

For the pristine samples, we fitted the *R vs. T* curves utilizing the Matthiessen rule of resistivity and including the Bloch-Gruneisen and the weak localization terms

$$\rho_{BG}(T) = C\left(\frac{T}{\theta_D}\right)^n \int_0^{\frac{T}{\theta_D}} \frac{x^n}{(e^x - 1)(1 - e^{-x})} dx$$

$n = 3$ for the case of s-d electron scattering in transition metals

$$\rho_{WL}(H,T) \sim b * \ln(T) - a * T^{1/2}$$

In Figure S9, we report representative fittings for two of the 1L and ML curves (Figure 2 of the manuscript). The model used to fit the data at low temperatures considers the 2D WL term $b * \ln(T)$ and the *e*-*e* interaction term $a * T^{1/2}$ at low temperatures, as often reported for disordered metallic systems [7,8]. From the fittings, we confirm a Bloch-Gruneisen temperature of ~ 680 K and ~ 635 K for the 1L and ML, respectively [9].

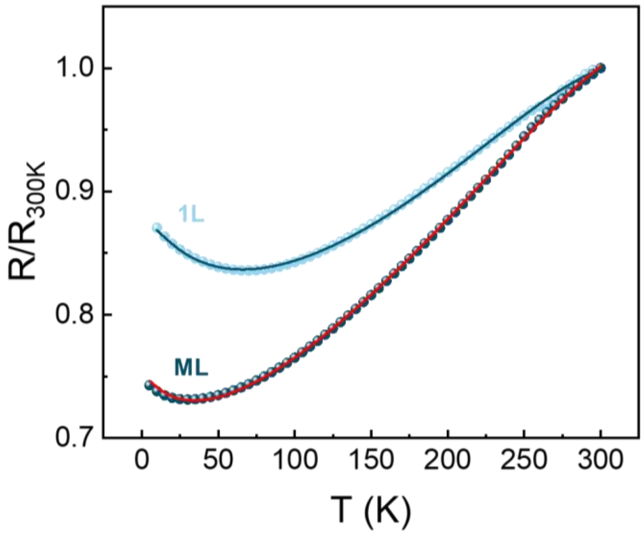

Figure S10. Resistance vs. temperature in monolayer and multi-layer device (spheres) with the relative fitting curves (solid lines).

8. *W*-function method and α evaluation

Hopping mechanisms are characterized by an exponential dependence of the conductivity σ on the temperature. A generic hopping mechanism can be modelled using the following equation

$$\sigma(T) = \sigma_0 \exp\left[-\left(\frac{T_0}{T}\right)^{\alpha}\right] = \frac{1}{\rho(T)}$$

with σ_0, T_0, and ρ representing the pre-factor, characteristic temperature, and resistivity of the system, respectively.

For α = 1, σ(*T*) is proportional to $\exp(E_a/k_B T)$, where $E_a = k_B T_0$ defines the activation energy of the nearest neighbor hopping (NNH) mechanism. On the contrary, all variable range hopping (VRH) mechanisms display α < 1, and the transport is ruled by charge carrier hopping between the two most energetically favorable sites, regardless of their spatial distance. To extract the right α coefficient and indicate the right hopping mechanism dominating the conduction, we can calculate the reduced activation energy (*W*)

$$W = -\frac{T}{\rho}\frac{\mathrm{d}\rho}{\mathrm{d}T}$$

Upon linearization of the previous equation, the slope of the ln(*W*) versus ln(*T*) plot returns the exponent α.

We applied this method to our system to definitely rule out hopping as the dominant charge transport mechanism in 600 °C annealed $Ti_3C_2T_x$ flakes.

Figure S11 shows the evaluated W for monolayer (a) and multilayer (b) devices.

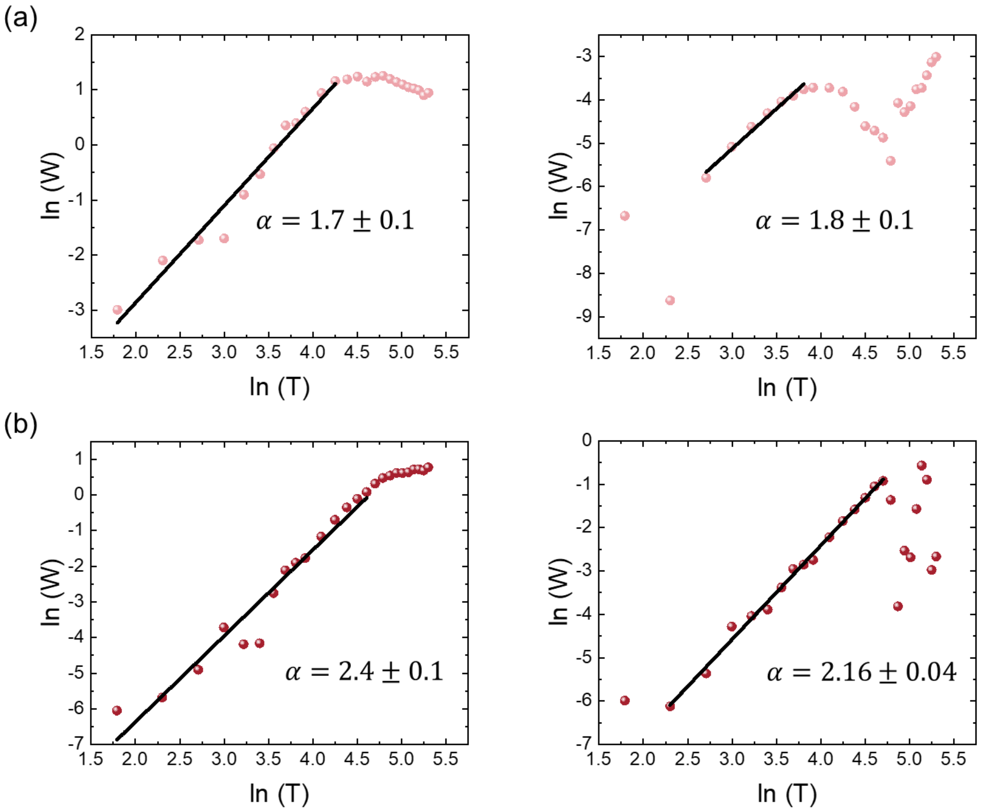

Figure S11. ln(W) versus ln(T) plots for two representative monolayers (a) and multilayers (b).

From the slopes of the curves in Figure S11, we can definitely conclude that the charge transport mechanism in the annealed samples cannot be attributed to any hopping. Monolayers show an average $\alpha = 1.8 \pm 0.4$, while $\alpha = 1.95 \pm 0.5$ for the multilayers. These α values do not correspond to any hopping mechanism; we have therefore proved that hopping cannot be considered as a valid charge transport mechanism in MXenes flakes.

9. Flake-to-flake variance

Since MXene is a solution-processed material, we considered the flake-to-flake variance in both pristine and annealed samples to statistically validate our data on the low-temperature upturn and metal to disordered metal transition upon annealing. Figure S12 displays all the possible scenarios upon 600 °C annealing, depending on the defectivity and disorder induced to the flakes.

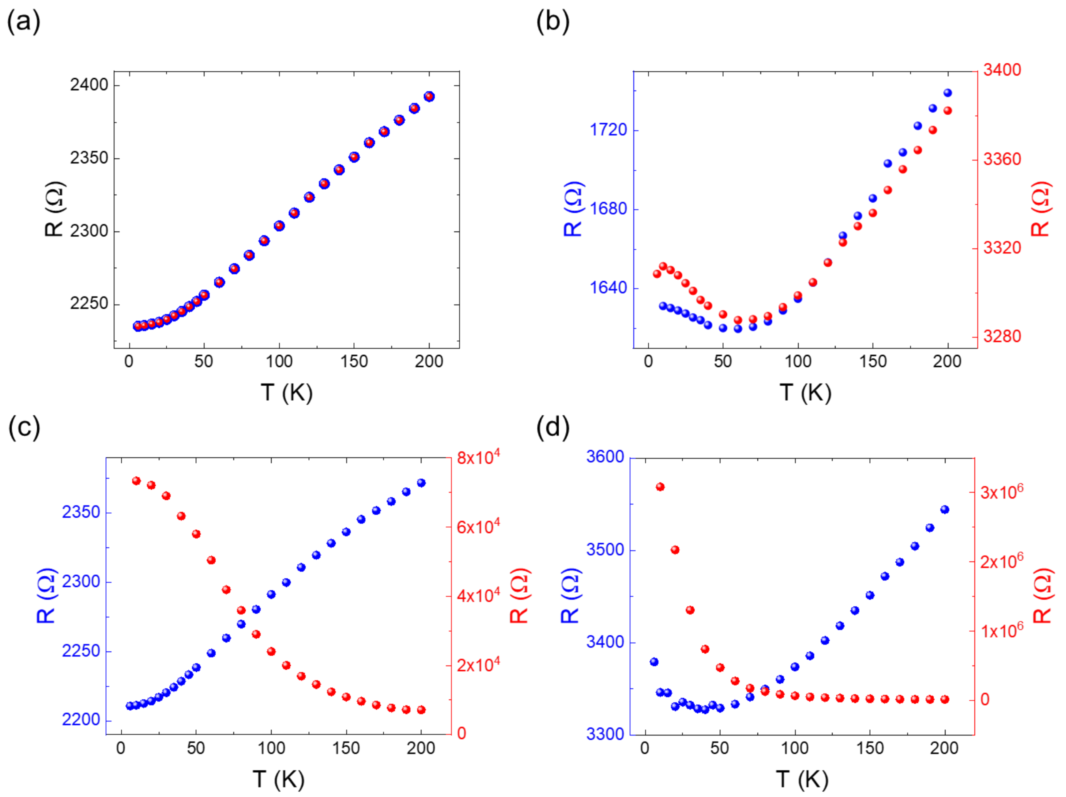

Figure S12. Flake-to-flake variation for 4 representative monolayer devices before (blue points) and after (red points) 600 °C annealing.

10. Mooij criterion

To better understand the deviation from the standard metallic behavior and find a correlation with the initial flake condition, we considered the Mooij criterion. The resistivity of metals varies with temperature, depending on both resistivity absolute value and disorder. When the resistivity is small, the material behaves in temperature affected by the electron-phonon scattering with a well-understood temperature dependence. For strongly defective systems, many models can describe the transport in the material; however, none of them alone works in the entire temperature range. Strongly disordered metals, including amorphous metals, metallic glasses, and alloys, show deviations from the standard metallic behavior, which can be attributed to the Mooij correlation, the saturation effect, and the breakdown of Matthiessen's rule. These may all be connected[10]. The size and sign of the temperature coefficient of resistivity ($\gamma = d\rho/dT$) in many disordered systems correlates well with their resistivity[10,11].

In Figure S13, we report the correlation between γ and ρ for the analyzed samples, with the exception of those showing ρ in the MΩ range and exponential behavior (for which the linear slope cannot be calculated).

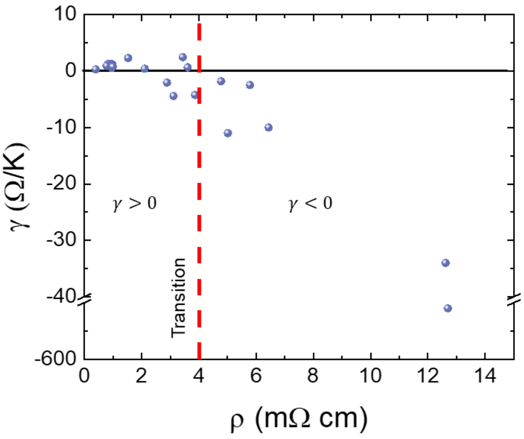

Figure S13. Mooij correlation criterion applied to the $Ti_3C_2T_x$ devices after 600°C annealing. The temperature coefficient γ is plotted as a function of the sample residual resistivity, displaying a transition from positive to negative in the range from 2 to 4 mΩ·cm.

Two representative curves showing the linear negative trend of the resistance in temperature are reported in Figure S14 for monolayer and multilayer, and corresponding linear fittings.

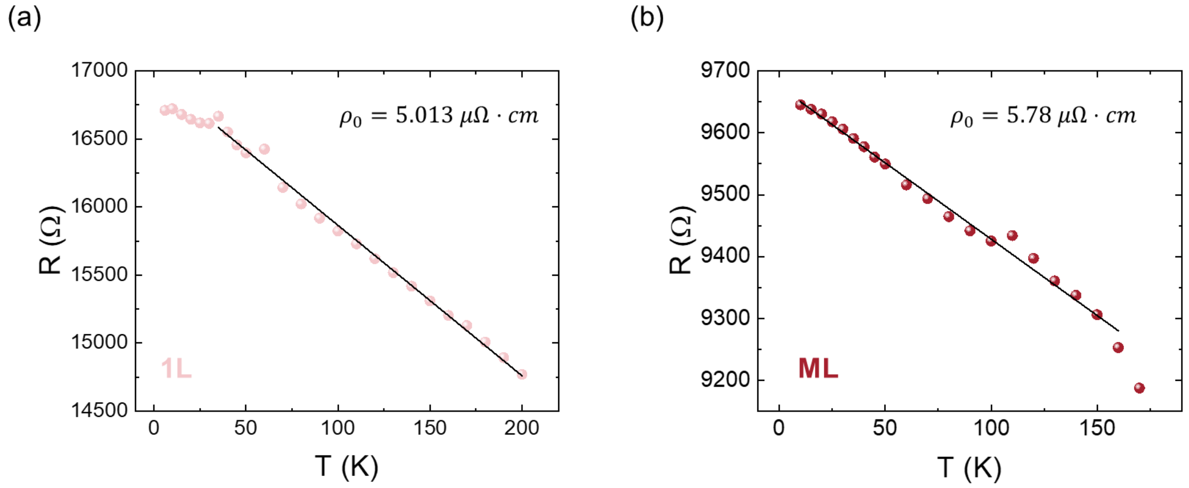

Figure S14. Monolayer and multilayer R vs T characteristics after 600°C annealing.

The Mooij criterion is an empirical relation between the R *vs.* T slope and the absolute value of the residual resistivity. In the original paper, above a threshold value of 150– 200 µm cm, its derivative (dρ/dT) changes sign from metallic (> 0) to non-metallic (< 0). However, such arguments have been developed for typical metallic systems and alloys, with carrier densities in the order of 10^{23} cm^3 [12]. $Ti_3C_2T_x$ is not a conventional metal, and as described by Belitz and Schirmacher, [13] the temperature dependence of the electrical resistivity in strongly disordered metals correlates with the charge carrier density in the metal, which in $Ti_3C_2T_x$ ranges from 10^{20} to 10^{21} cm^3 [14]. We believe that this property could explain the higher resistivity threshold value, considering that the compound is already described by higher resistivities in its pristine state.

11. I-V curves before and after temperature annealing

Room-temperature I-V curves of pristine and annealed samples were recorded to prove the stability of the contacts upon annealing. Ohmic contacts at 300 K are observed during both sets of measurements, as shown by the plots in Figure S15.

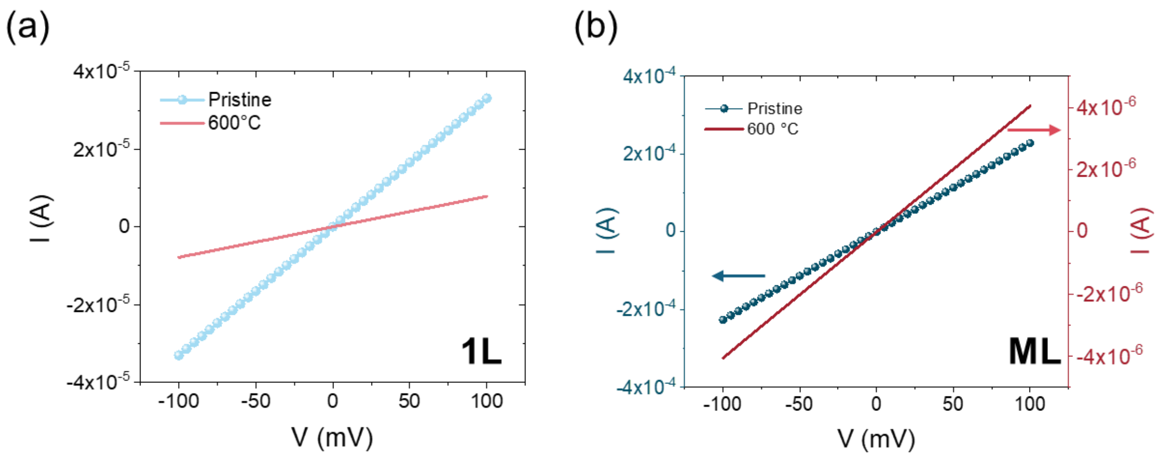

Figure S15. I-V characteristics (at room temperature) for three representative samples (a) mono-, (b) multi-layer, before and after the 600 °C annealing.

12. Magnetoresistance analysis

The magnetoresistance (MR) and angular-dependent MR measurements were performed in a Quantum Design PPMS using a horizontal rotator. The fittings of the magnetotransport data with a weak localization (WL) model were performed using Mathematica. The experimental data points and the fitting curves for different devices are reported in Figure S16.

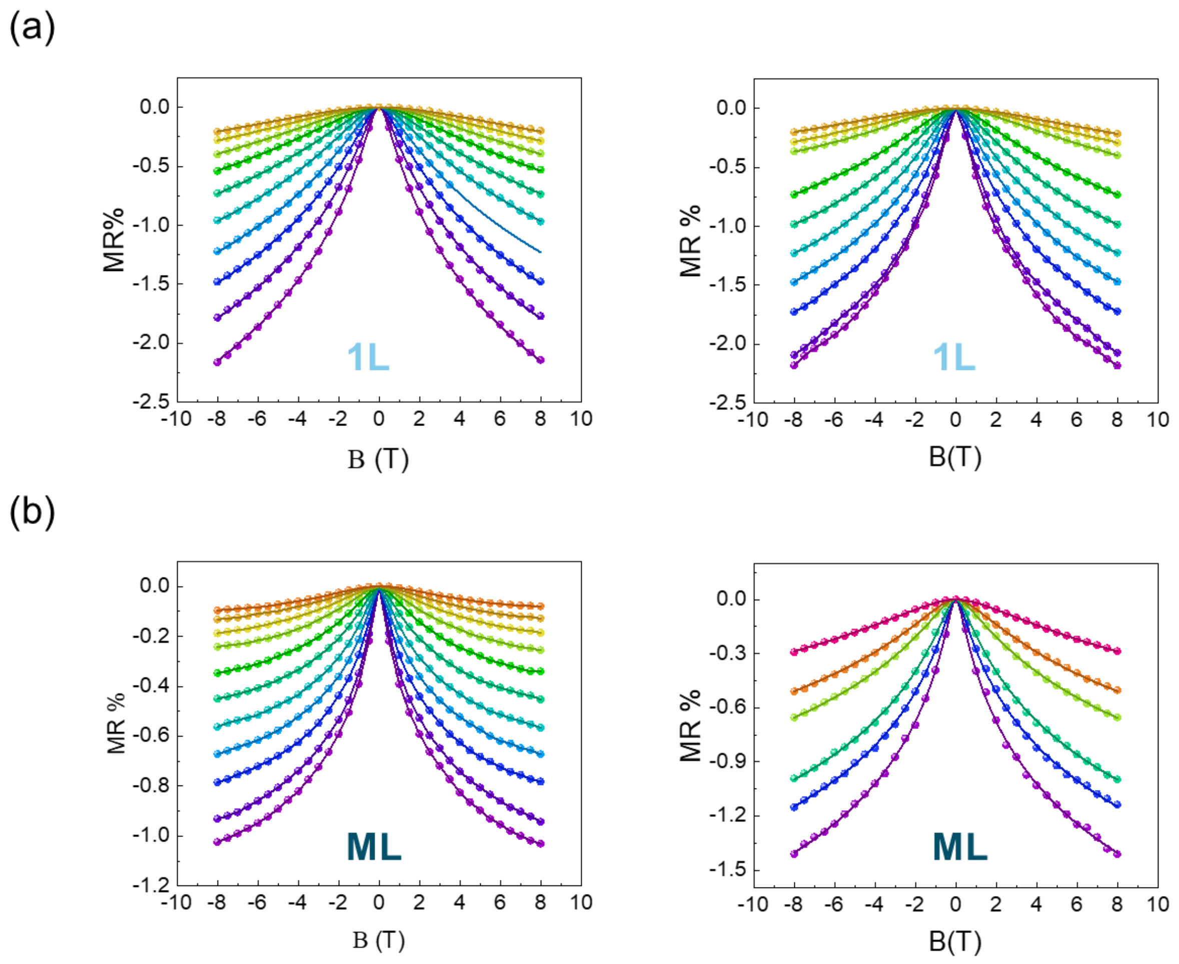

Figure S16. Field-dependent MR for (a) monolayer and (b) multi-layer pristine devices. The solid lines represent the fitting curve obtained from the Hikami-Larkin-Nagaoka theory.

To fit the MR *vs.* $\mu_0 H$ curves, we utilized the Hikami-Larkin-Nagaoka model[15]. To avoid overparameterization of the problem, we minimized the number of free parameters, considering the expression for diffusive metals with high spin-orbit coupling ($\tau_\phi \gg \tau_{SO}, \tau_e$), where τ_ϕ is the phase coherence or dephasing time, τ_{SO} and τ_e are the spin-orbit and elastic scattering time, respectively.

The model equation can, therefore, be written as follows:

$$\mathrm{MR} = -\alpha \frac{e^2}{\pi h}\left[\psi\left(\frac{1}{2} + \frac{B_\phi}{|B|}\right) + \ln \frac{B_\phi}{|B|}\right]$$

where α is an empirical fitting parameter and B_ϕ represents the phase coherence field. Our estimated α is positive, ranging from 1.4 to 2, which agrees with values reported for other 2D systems [16,17]. From the obtained values of B_ϕ, we calculated the phase coherence length L_ϕ. The trend of the L_ϕ as a function of temperature, is shown in Figure S17 below, for devices of different thicknesses.

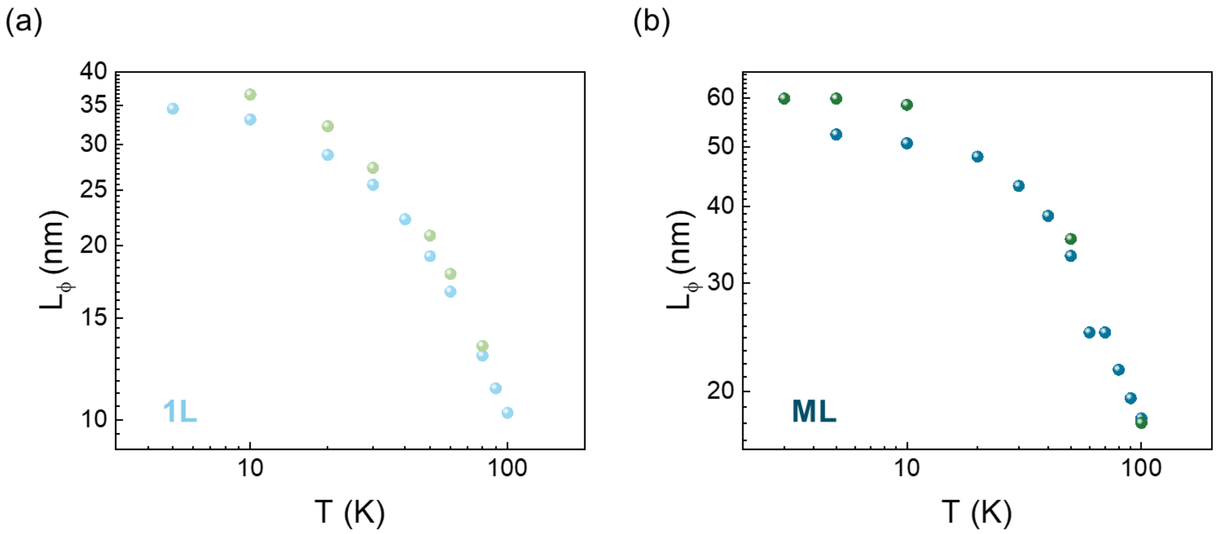

Figure S17. Phase coherence lengths as a function of temperature for (a) 1L samples and (b) ML samples. The $T^{-0.5}$ behavior at temperatures above 20 K can be recognized.

To generalize our results and provide correct and compelling claims, we based our work on strong device statistics; therefore we fabricated and analyzed ~30 single-flake devices. As reported in Figure S17, the phase coherence lengths are considered on average on a couple of devices for both single- and multi-layers.

13. Magnetoresistance measurements after thermal treatment

We conducted the magnetoresistance measurements after thermal treatment at 600 °C. Given the high resistance of the sample and the absence of weak localization signatures, the MR does not vary with the applied field, showing a scattered behavior around zero (Figure S18).

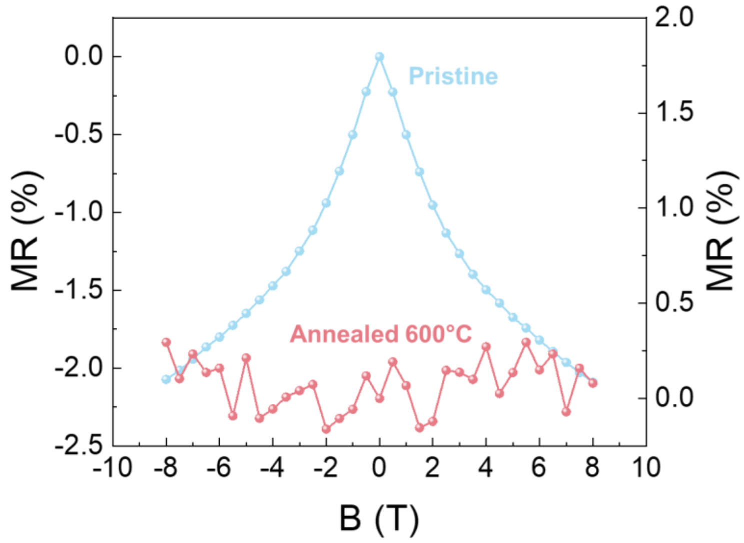

Figure S18 Comparison of the MR as a function of the applied magnetic field for the pristine (blue) and 600 °C annealed (red) flake.

14. Secondary ion mass spectrometry

All secondary ion mass spectroscopy (SIMS) experiments were performed with the CAMECA IMS SC Ultra instrument equipped with a cesium source. As previously described,[18] several modifications of the measurement procedure are required to ensure atomic depth resolution: high incident angle bombardment (75°), ultra-low impact energy (100 eV), *in-situ* ion polishing, optimization of extraction parameters, super cycle, and advanced beam positioning. Additionally, a deconvolution and calibration protocol[19] was applied to quantify the results and determine the exact composition of each atomic layer. The SIMS profiles for pristine and annealed MXenes (1L and ML) are displayed in Figure S19.

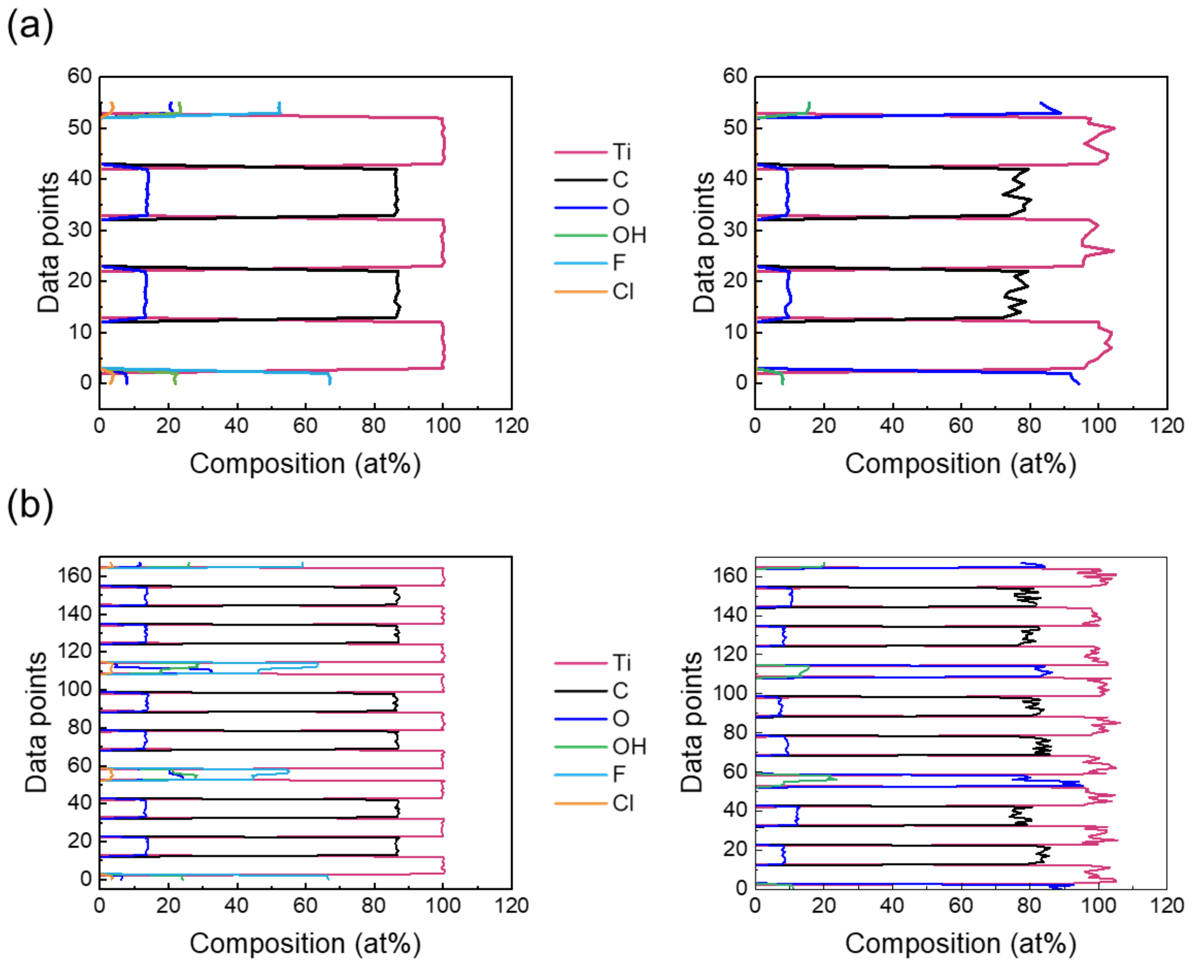

Figure S19. SIMS measurements were performed on (a) 1L and (b) ML pristine (left panels) and annealed (right panels) devices.

The annealed devices show noisier signals, with wide fluctuations and different atomic concentrations in the C, Ti, and O layers, compared to the pristine samples. We attribute this to an increased level of structural defectivity introduced in the material by the annealing process, which led to the compositional change of the termination layers, the release of some carbon and oxygen from the X layers, and the general disorder and defect degradation of the structure (both in the X and M layers). To corroborate our attribution, we measured the depth profile of a $Ti_3C_2T_x$ sample bombarded with a high-energy cesium beam to deliberately introduce

defects in the structure. For this profile (Figure S20), the noise level increases considerably; thus, by comparing it with the annealed samples, we conclude that the noise level indirectly informs us about the sample quality (structural defectivity).

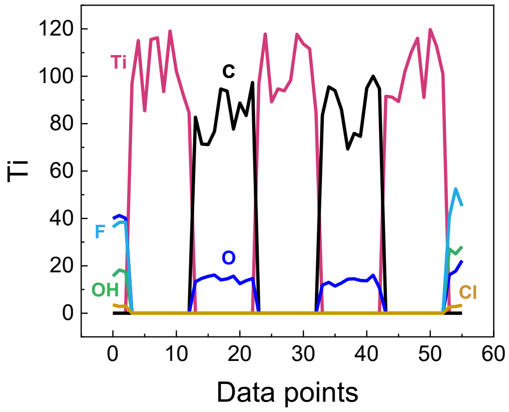

Figure S20. The SIMS depth profile of a single-flake MXene sample was implanted with a highly energetic cesium beam (15 keV) with a very low dose (5×10^{12} ions/cm^2). For such a dose, less than 1% of the sample surface was impacted; thus, the overall layered structure remains intact. However, some structural changes (broken bonds, atom misplacements, etc.) are expected, and apparently, their presence introduces a high noise to the SIMS experiments. Thus, the noise level can be indirectly used to monitor the quality of a sample.

15. Density functional theory calculations

The density functional theory (DFT) calculations were performed using the VASP package[20] and, unless stated otherwise, the PBE exchange-correlation functional[21,22]. The MXene models used are periodic in all lattice vectors, and the plane-wave formalism[20] was used to describe the electronic wave functions. The cutoff energy for the plane waves was set at 550 eV for unit cell size optimization and 415 eV for supercell calculations. The force and energy convergence criteria were set to 0.005 eV/Å (0.01 eV/Å) and 10^{-6} eV (10^{-5} eV), respectively, for unit cell (supercell) calculations. Brillouin zone sampling was done using sets of 15×15×1 (3×3×1) Gamma-centered special k points for unit cell (supercell) calculations[23]. The band diagrams were obtained using an explicit list of 120 k points interpolated between the high-symmetry Γ-M-K-Γ path. To mitigate unwanted interaction between periodic replicas of the two-dimensional model system in the direction perpendicular to the surface plane, 15 Å of vacuum width was included in the simulation boxes. The $Ti_3C_2T_x$ models containing atomic defects were built from supercells containing 4×4 MXene unit cells, leading to approximately 12 Å of separation between single-atom defects.

It is known that $Ti_3C_2T_x$ is metallic regardless of the chosen functionalization groups[24,25]. Indeed, that is what our calculations predict for bare Ti_3C_2 and $Ti_3C_2O_2$, as shown by the band diagrams at the top of Figure S21. Since the PBE functional is known to underestimate the band gaps of materials, we recalculated the band diagrams with the HSE06 hybrid functional[26,27] for the unit cells only (because of the huge computational cost of such calculations on large periodic supercells). In the case of MXenes, using HSE06 instead of PBE to calculate band structures has been reported to yield the same shape of the valence and conduction bands but

more widely separated,[28–30] an effect that can be seen in the bottom panels of Figure S21. Although HSE06 does seem to increase the spacing between bands, it does not predict the opening of a band gap at the Fermi level. The PBE-level band diagrams of Ti_3C_2 and $Ti_3C_2O_2$ MXene unit cells shown in Figure S21 coincide with the ones calculated at the same level of theory in Refs.[31,32], respectively.

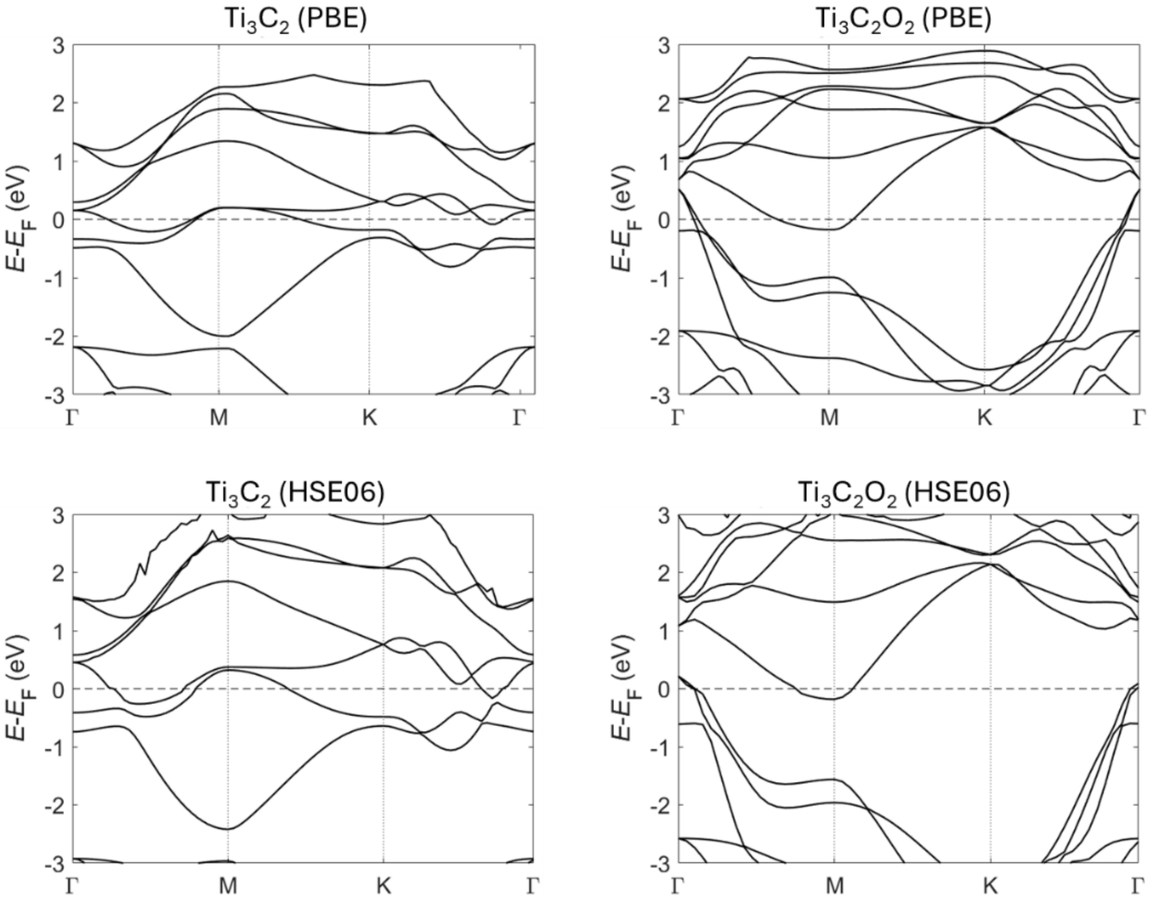

Figure S21. PBE (top) and HSE06 (bottom) band diagrams of the bare Ti_3C_2 (left) and $Ti_3C_2O_2$ (right) MXene unit cells.

We further focused on Ti vacancies and their effects on the electronic band-structure. To study how the inclusion of Ti vacancies at different densities affects the band structure, we used the larger supercells containing 4×4 MXene unit cells, deleted the appropriate number of Ti atoms of one of the outer layers, relaxed the atomic positions, and then calculated the formation energy per Ti vacancy and the respective band diagram. The formation energy per Ti vacancy of a MXene model containing n Ti vacancies is given by

$$E_{\mathrm{form}} = \frac{E(\mathrm{MXene} - n\ \mathrm{Ti}) - E(\mathrm{MXene}) - n\, E(\mathrm{Ti})}{n}$$

where $E(\mathrm{MXene})$ is the total energy of the relaxed MXene model before Ti vacancies are introduced, $E(\mathrm{MXene} - n\ \mathrm{Ti})$ is the total energy (after relaxation) of the supercell with n Ti vacancies, and $E(\mathrm{Ti})$ is the energy per atom of a bulk Ti unit cell. The calculated formation energies are given in Table S1.

Table S1. Calculated formation energies per Ti vacancy, in eV, for Ti_3C_2 and $Ti_3C_2O_2$ MXenes.

n	Ti vacancy concentration (at%)	Ti_3C_2	$Ti_3C_2O_2$
1	2	2.68	7.72
2	4	2.69	7.98
4	8	2.68	8.93
8	17	2.64	8.81

Here, we focused on Ti vacancies present on the outermost Ti layers of the MXene models. While the formation energy of one Ti vacancy on an outer Ti layer of Ti_3C_2 is 2.68 eV, that of a vacancy in the inner Ti layer is 6.14 eV, i.e., almost 3.5 eV higher. Note that this difference is independent of the reference considered for the energy of a single Ti atom in the calculation of the formation energies. Furthermore, we calculated the migration barrier of a Ti vacancy along the surface of the Ti_3C_2 MXene using the climbing image nudged elastic band method[33,34], and found a value of 2.54 eV. Additionally, the energy barrier for a Ti atom in the inner layer to occupy a Ti vacancy on the surface layer, passing through the middle C layer and creating a new Ti vacancy in the inner Ti layer, is 4.35 eV, whereas the reverse process involves a barrier of only 1.08 eV[35]. Therefore, Ti vacancies on the surface should be more likely to travel along the plane of the surface than to be filled by Ti atoms coming from the inner Ti layer.

It is noteworthy that the Ti vacancy formation energies on $Ti_3C_2O_2$ are roughly three times higher than in Ti_3C_2, in agreement with the results in Ref.[36], where the calculated formation energies of a single Ti vacancy on Ti_3C_2 and $Ti_3C_2O_2$ were 2.85 and 7.74 eV, respectively. For comparison, the calculated formation energies of a C vacancy are 2.61 and -0.36 eV for Ti_3C_2 and $Ti_3C_2O_2$, respectively. The thermodynamically favorable creation of C vacancies in $Ti_3C_2O_2$ has already been predicted in the literature[35,37]. Thus, at least from the thermodynamic point of view, it is reasonable to have both C and Ti vacancies in $Ti_3C_2T_x$. Note that all formation energies are calculated with respect to some reference species. In this case, the energy of a Ti atom was calculated as the energy per atom of a bulk Ti unit cell, and the energy of a C atom as the energy per atom of a diamond unit cell. In principle, the reference species can be selected to change the difference between the formation energies of Ti and C at will.

Experimentally, the samples were analyzed after annealing and before exposure to air, implying that a large fraction of the surface termination has been removed, greatly facilitating the creation of Ti vacancies. Moreover, the subsurface substitutional O atoms and the creation of O and C vacancies, as evidenced from the SIMS analysis, in the vicinity of the Ti atoms, reduces the energy formation of Ti vacancies and misplacement. The former have been known to occur since the identification of oxycarbides[18,38], while the latter were observed in the present work, namely about 14.1 at% of vacancies in the X layers of the MXene. Here, we obtained a

single Ti vacancy E_{form} of 1.68 eV near a C vacancy on Ti_3C_2, which is 1 eV lower than on the pristine material. Moreover, on $Ti_3C_2O_2$, the E_{form} energy of a single Ti vacancy is reduced from 7.72 eV to 6.40 eV if there is one O vacancy next to the Ti atom, to 4.64 eV if there are two, and to 2.54 eV if near three O vacancies. Nevertheless, the greatest reduction in the formation energy for Ti vacancies is found on the $Ti_3C_{2-x}O_x$ oxycarbide MXene; when near a subsurface O atom, the Ti vacancy formation energy is reduced to 1.65 eV, and with 25 % of the C atoms replaced by O ones, it is further reduced to 1.51 eV.

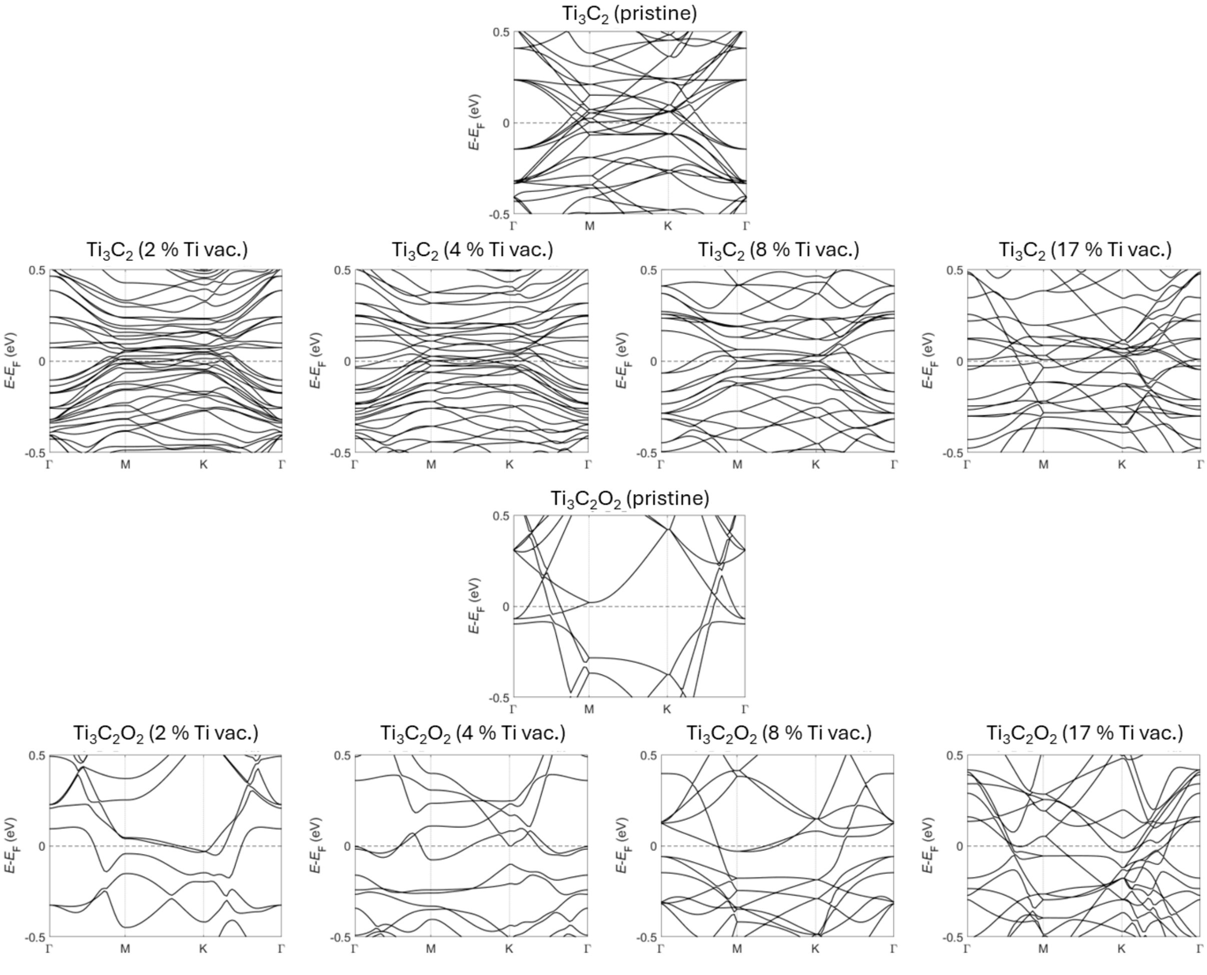

Figure S22. Calculated band diagrams of the Ti_3C_2 (top) and $Ti_3C_2O_2$ (bottom) MXene models with different Ti vacancy concentrations for the 4×4 supercell.

The calculated band diagrams corresponding to the eight systems of Table 1 are displayed in Figure S22. Note that the seemingly increased number of bands in the plots of Figure S22, when compared to the ones in Figure S21, is in fact due to the smaller Brillouin zone of larger periodic cells. Since the band diagrams of Figure S22 correspond to supercells with 4×4 unit cells, the physical meaning of the band diagrams of the pristine material remains the same, but band folding occurs, as shown, for example, in Ref.[39] for Ti_2CO_2 MXene supercells with different sizes. Clearly, Ti vacancies in any reasonable concentration do not turn the material into a semiconductor. Given the typical underestimation of band gaps by the PBE functional, we performed single-point calculations on the PBE-relaxed structures using the HSE06 hybrid functional and automatic grids of

special k points. This procedure was chosen because reducing the density of k points should yield a band gap greater than or equal to the one found with a denser sampling. Therefore, knowing that our HSE06 calculation with fewer k points predicted metallicity in all situations, then using more k points would predict the same behavior.

16. Scanning electron microscopy and energy dispersive spectroscopy

We performed EDS mappings (Figure S23) on $Ti_3C_2T_x$ single-flake devices to confirm the homogeneous elemental distributions.

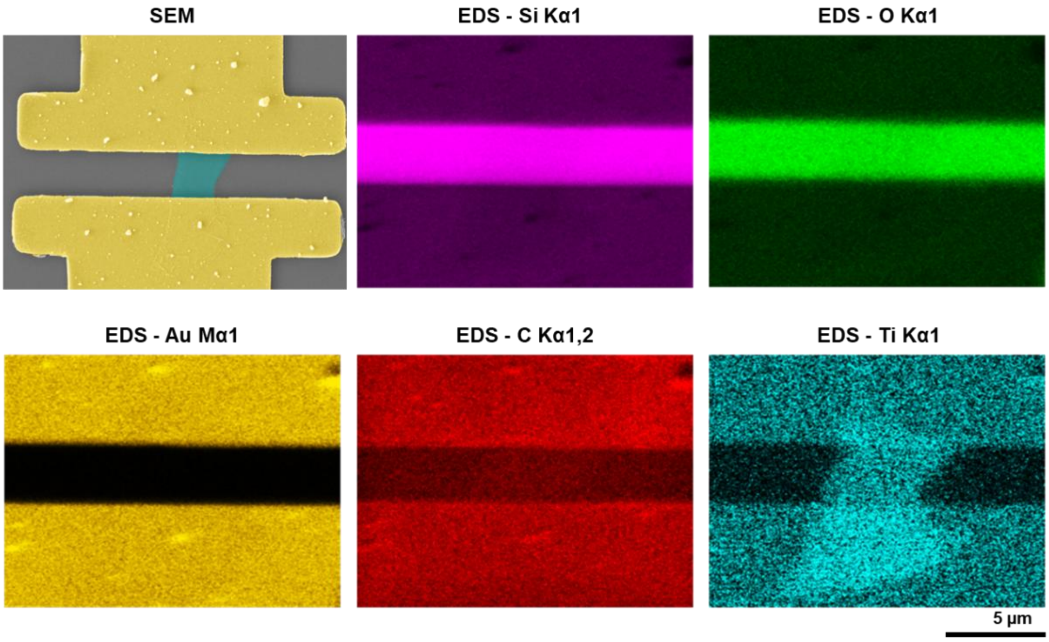

Figure S23. False-colored SEM image and related EDS elemental mapping analysis for a typical $Ti_3C_2T_x$ device.

17. Transmission electron microscopy

From the transmission electron microscopy (TEM) images, we observe the appearance of a "patchy" texture after annealing at 600 °C. From the electron diffraction pattern on pristine and annealed flakes (Figure S24 right panels), we can attribute such a contrast difference in panel (b) left, to the different local crystal orientation or to the formation of locally thicker and thinner areas due to the Ti and C dislocation[40,41].

The right panel (b) shows how the crystal electron diffraction pattern deviates from the periodic hexagonal arrangement of the pristine $Ti_3C_2T_x$ (panel (a) right).

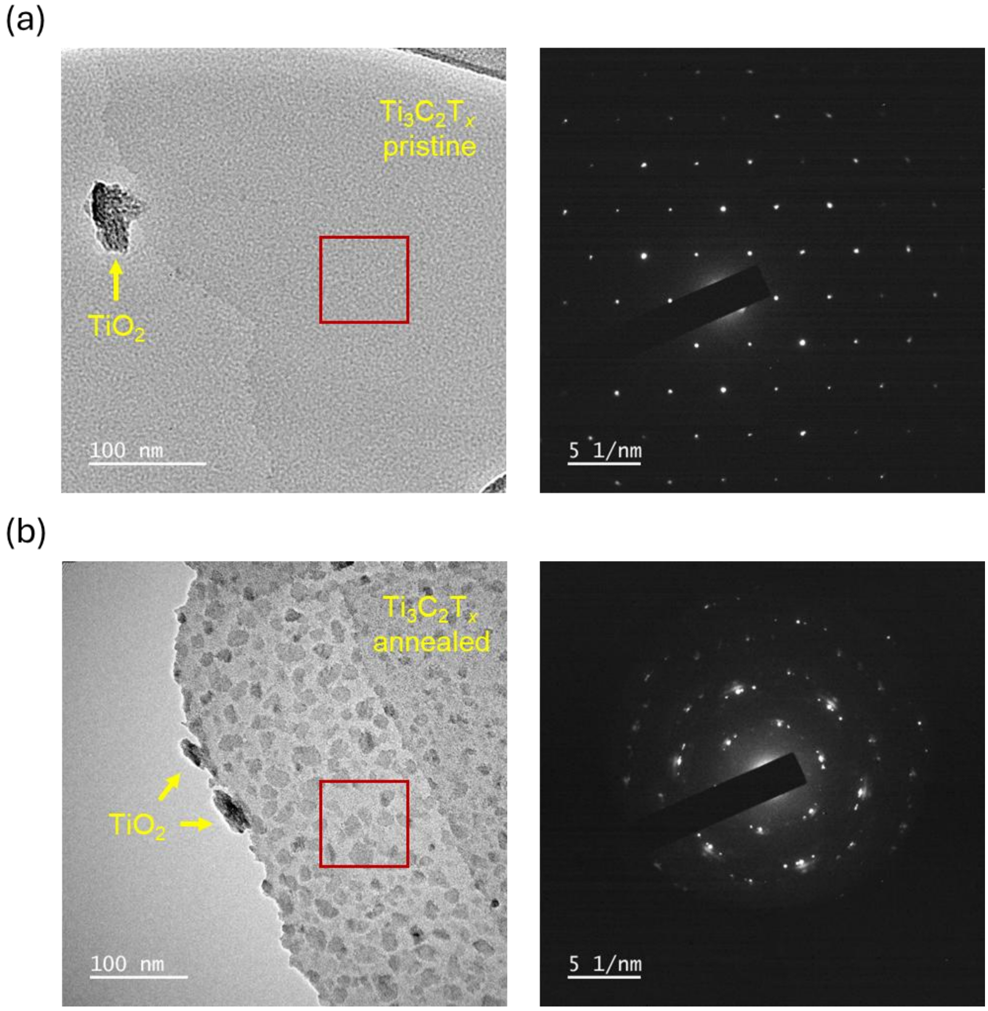

Figure S24. TEM images (left) and electron diffraction patterns (right) of the pristine (a) and 600 °C annealed (b) flake.

If TiO_2 were present, intense rings would appear in the green and blue regions (typical for TiO_2) in Figure S24. However, upon thermal treatment, only additional diffraction spots appear (with distance values analogous for pristine and annealed samples), rather than intense rings characteristic of TiO_2, further corroborating the lack of oxidation. Such additional new diffraction spots (not intense rings) arise after treatment, which well correlate with the increase of disorder and defectivity within the lattice.

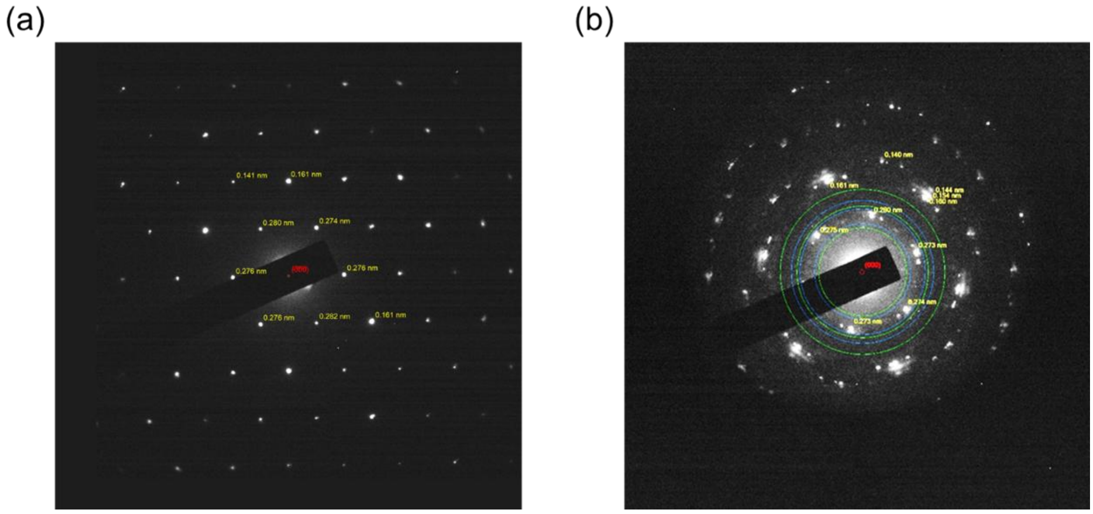

Figure S25. Electron diffraction patterns and resolved spacing of the pristine (a) and 600 °C annealed (b) flake. The green and blue rings correspond to where TiO_2 (in both rutile and anatase phase) highest intensity peaks would be, ruling out oxidation.

From the TEM images of Figures S24 and S25 we can further exclude the presence of oxide formation during the 600°C annealing. The figure below displays the TEM images taken at different magnifications for a single layer after 600 °C annealing.

These results confirm what previously reported in the literature[42,43]. Through X-ray Photoelectron Spectroscopy (XPS) analysis the authors prove that the thermal annealing in ultra-high vacuum up to 750 °C does not affect the $Ti_3C_2T_x$ stability and does not induce oxidation to TiO_2. In particular, the XPS spectra, recorded at different temperatures, along with the relative STEM images, confirm the lack of oxidation upon thermal treatment [42]. In addition, as reported by Fang et al.[43] the temperature annealing at 600°C stabilizes the material (by removing confined water) and avoids oxidation of the MXene flakes during the following annealing in air.

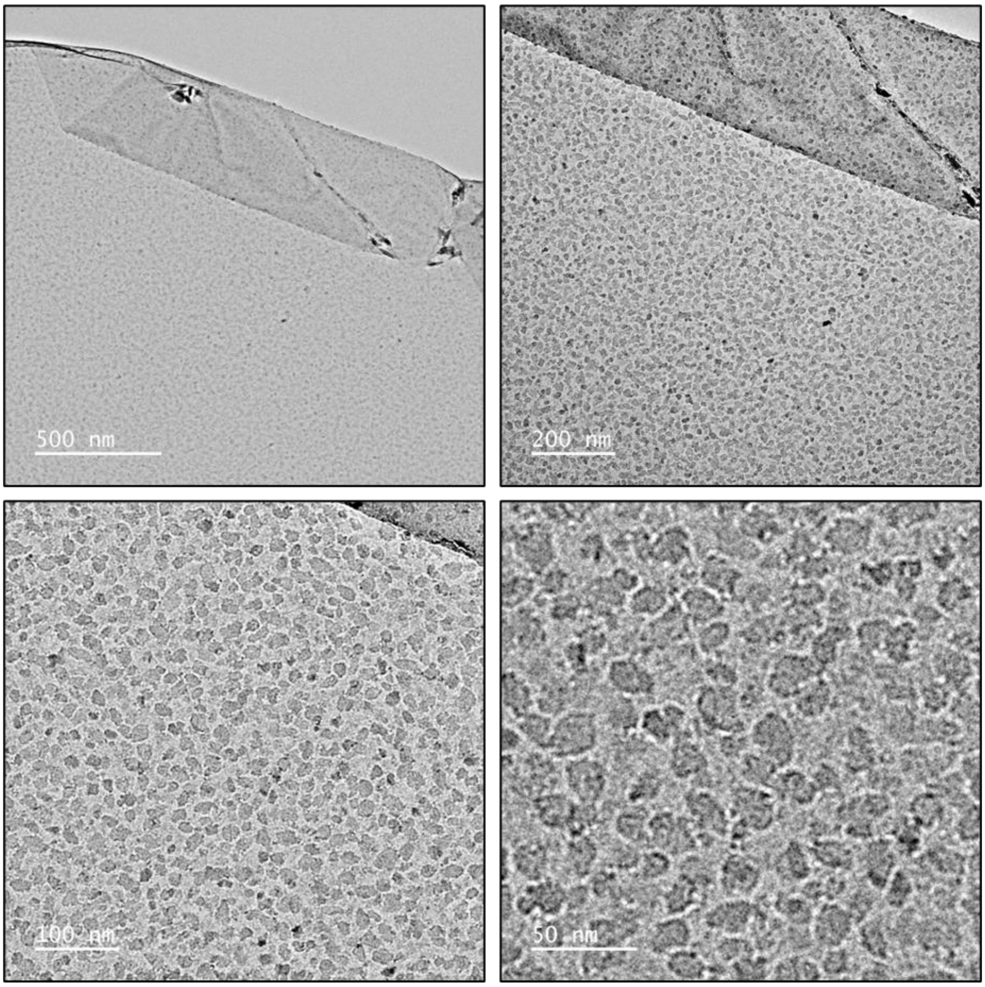

Figure S26. TEM images of the 600 °C annealed flake at different magnifications.

18. References

(1) Downes, M.; Shuck, C. E.; McBride, B.; Busa, J.; Gogotsi, Y. Comprehensive Synthesis of $Ti_3C_2T_x$ from MAX Phase to MXene. *Nat. Protoc.* **2024**, *19* (6), 1807–1834. https://doi.org/10.1038/s41596-024-00969-1.

(2) Sarycheva, A.; Gogotsi, Y. Raman Spectroscopy Analysis of the Structure and Surface Chemistry of $Ti_3C_2T_x$ MXene. *Chem. Mater.* **2020**, *32* (8), 3480–3488. https://doi.org/10.1021/acs.chemmater.0c00359.

(3) Mignuzzi, S.; Pollard, A. J.; Bonini, N.; Brennan, B.; Gilmore, I. S.; Pimenta, M. A.; Richards, D.; Roy, D. Effect of Disorder on Raman Scattering of Single-Layer Mo S 2. *Phys. Rev. B* **2015**, *91* (19), 195411. https://doi.org/10.1103/PhysRevB.91.195411.

(4) Ferrari, A. C.; Basko, D. M. Raman Spectroscopy as a Versatile Tool for Studying the Properties of Graphene. *Nature Nanotech* **2013**, *8* (4), 235–246. https://doi.org/10.1038/nnano.2013.46.

(5) Plaickner, J.; Petit, T.; Bärmann, P.; Schultz, T.; Koch, N.; Esser, N. Surface Termination Effects on Raman Spectra of $Ti_3C_2T_x$ MXenes: An *in Situ* UHV Analysis. *Phys. Chem. Chem. Phys.* **2024**, *26* (31), 20883–20890. https://doi.org/10.1039/D4CP02197E.

(6) Boschi, A.; Kovtun, A.; Liscio, F.; Xia, Z.; Kim, K. H.; Avila, S. L.; De Simone, S.; Mussi, V.; Barone, C.; Pagano, S.; Gobbi, M.; Samorì, P.; Affronte, M.; Candini, A.; Palermo, V.; Liscio, A. Mesoscopic 3D Charge Transport in Solution-Processed Graphene-Based Thin Films: A Multiscale Analysis. *Small* **2023**, *19* (42), 2303238. https://doi.org/10.1002/smll.202303238.

(7) Thompson, R. S.; Li, D.; Witte, C. M.; Lu, J. G. Weak Localization and Electron−Electron Interactions in Indium-Doped ZnO Nanowires. *Nano Lett.* **2009**, *9* (12), 3991–3995. https://doi.org/10.1021/nl902152c.

(8) Gumbatov, S. G.; Pashaev, Kh. M.; Panova, G. Kh.; Shikov, A. A. Weak Electron Localization and Electron-Electron Interaction Effects in Metallic Glasses at Temperatures Just above Superconducting Transition. *Solid State Communications* **1986**, *58* (6), 389–392. https://doi.org/10.1016/0038-1098(86)90811-2.

(9) Lipatov, A.; Bagheri, S.; Sinitskii, A. Metallic Conductivity of $Ti_3C_2T_x$ MXene Confirmed by Temperature-Dependent Electrical Measurements. *ACS Mater. Lett.* **2024**, *6* (1), 298–307. https://doi.org/10.1021/acsmaterialslett.3c01234.

(10) Lee, P. A.; Ramakrishnan, T. V. Disordered Electronic Systems. *Rev. Mod. Phys.* **1985**, *57* (2), 287–337. https://doi.org/10.1103/RevModPhys.57.287.

(11) Mooij, J. H. Electrical Conduction in Concentrated Disordered Transition Metal Alloys. *Phys. Stat. Sol. (a)* **1973**, *17* (2), 521–530. https://doi.org/10.1002/pssa.2210170217.

(12) Martino, E.; Arakcheeva, A.; Autès, G.; Pisoni, A.; Bachmann, M. D.; Modic, K. A.; Helm, T.; Yazyev, O. V.; Moll, P. J. W.; Forró, L.; Katrych, S. Sr_2Pt_{8-x} As: A Layered Incommensurately Modulated Metal with Saturated Resistivity. *IUCrJ* **2018**, *5* (4), 470–477. https://doi.org/10.1107/S2052252518007303.

(13) Belitz, D.; Schirmacher, W. Theory of Phonon-Controlled Conductivity in High-Resistivity Conductors. *J. Phys. C: Solid State Phys.* **1983**, *16* (5), 913–926. https://doi.org/10.1088/0022-3719/16/5/018.

(14) Miranda, A.; Halim, J.; Barsoum, M. W.; Lorke, A. Electronic Properties of Freestanding $Ti_3C_2T_x$ MXene Monolayers. *Appl. Phys. Lett.* **2016**, *108* (3), 033102. https://doi.org/10.1063/1.4939971.

(15) Hikami, S.; Larkin, A. I.; Nagaoka, Y. Spin-Orbit Interaction and Magnetoresistance in the Two Dimensional Random System. *Prog. Theor. Phys.* **1980**, *63* (2), 707–710. https://doi.org/10.1143/ptp.63.707.

(16) Papadopoulos, N.; Watanabe, K.; Taniguchi, T.; Van Der Zant, H. S. J.; Steele, G. A. Weak Localization in Boron Nitride Encapsulated Bilayer MoS2. *Phys. Rev. B* **2019**, *99* (11), 115414. https://doi.org/10.1103/physrevb.99.115414.

(17) Neal, A. T.; Liu, H.; Gu, J.; Ye, P. D. Magneto-Transport in MoS_2: Phase Coherence, Spin–Orbit Scattering, and the Hall Factor. *ACS Nano* **2013**, *7* (8), 7077–7082. https://doi.org/10.1021/nn402377g.

(18) Michałowski, P. P.; Anayee, M.; Mathis, T. S.; Kozdra, S.; Wójcik, A.; Hantanasirisakul, K.; Jóźwik, I.; Piątkowska, A.; Możdżonek, M.; Malinowska, A.; Diduszko, R.; Wierzbicka, E.; Gogotsi, Y. Oxycarbide MXenes and MAX Phases Identification Using Monoatomic Layer-by-Layer Analysis with Ultralow-Energy Secondary-Ion Mass Spectrometry. *Nat. Nanotechnol.* **2022**, *17* (11), 1192–1197. https://doi.org/10.1038/s41565-022-01214-0.

(19) Michałowski, P. P. Unraveling the Composition of Each Atomic Layer in the MXene/MAX Phase Structure – Identification of Oxycarbide, Oxynitride, and Oxycarbonitride Subfamilies of MXenes. *Nanoscale Horiz.* **2024**, *9* (9), 1493–1497. https://doi.org/10.1039/D4NH00151F.
(20) Kresse, G.; Furthmüller, J. Efficient Iterative Schemes for *Ab Initio* Total-Energy Calculations Using a Plane-Wave Basis Set. *Phys. Rev. B* **1996**, *54* (16), 11169–11186. https://doi.org/10.1103/PhysRevB.54.11169.
(21) Mak, K. F.; McGill, K. L.; Park, J.; McEuen, P. L. The Valley Hall Effect in MoS_2 Transistors. *Science* **2014**, *344* (6191), 1489–1492. https://doi.org/10.1126/science.1250140.
(22) Perdew, J. P.; Burke, K.; Ernzerhof, M. Generalized Gradient Approximation Made Simple. *Phys. Rev. Lett.* **1996**, *77* (18), 3865–3868. https://doi.org/10.1103/PhysRevLett.77.3865.
(23) Monkhorst, H. J.; Pack, J. D. Special Points for Brillouin-Zone Integrations. *Phys. Rev. B* **1976**, *13* (12), 5188–5192. https://doi.org/10.1103/PhysRevB.13.5188.
(24) Bai, Y.; Zhou, K.; Srikanth, N.; Pang, J. H. L.; He, X.; Wang, R. Dependence of Elastic and Optical Properties on Surface Terminated Groups in Two-Dimensional MXene Monolayers: A First-Principles Study. *RSC Adv.* **2016**, *6* (42), 35731–35739. https://doi.org/10.1039/C6RA03090D.
(25) Faraji, M.; Bafekry, A.; Fadlallah, M. M.; Molaei, F.; Hieu, N. N.; Qian, P.; Ghergherehchi, M.; Gogova, D. Surface Modification of Titanium Carbide MXene Monolayers (Ti_2C and Ti_3C_2) *via* Chalcogenide and Halogenide Atoms. *Phys. Chem. Chem. Phys.* **2021**, *23* (28), 15319–15328. https://doi.org/10.1039/D1CP01788H.
(26) Heyd, J.; Scuseria, G. E.; Ernzerhof, M. Hybrid Functionals Based on a Screened Coulomb Potential. *The Journal of Chemical Physics* **2003**, *118* (18), 8207–8215. https://doi.org/10.1063/1.1564060.
(27) Krukau, A. V.; Vydrov, O. A.; Izmaylov, A. F.; Scuseria, G. E. Influence of the Exchange Screening Parameter on the Performance of Screened Hybrid Functionals. *The Journal of Chemical Physics* **2006**, *125* (22), 224106. https://doi.org/10.1063/1.2404663.
(28) Bae, S.; Kang, Y.-G.; Khazaei, M.; Ohno, K.; Kim, Y.-H.; Han, M. J.; Chang, K. J.; Raebiger, H. Electronic and Magnetic Properties of Carbide MXenes—the Role of Electron Correlations. *Materials Today Advances* **2021**, *9*, 100118. https://doi.org/10.1016/j.mtadv.2020.100118.
(29) Gouveia, J. D.; Gomes, J. R. B. Effect of Surface Composition on the Stability of Ti- and V-Based Oxycarbide and Oxynitride MXenes. *Materials Today Physics* **2024**, *46*, 101481. https://doi.org/10.1016/j.mtphys.2024.101481.
(30) Xie, Y.; Kent, P. R. C. Hybrid Density Functional Study of Structural and Electronic Properties of Functionalized $Ti_{n+1}X_n$ (X = C , N) Monolayers. *Phys. Rev. B* **2013**, *87* (23), 235441. https://doi.org/10.1103/PhysRevB.87.235441.
(31) Yorulmaz, U.; Demiroğlu, İ.; Çakir, D.; Gülseren, O.; Sevik, C. A Systematical Ab-Initio Review of Promising 2D MXene Monolayers towards Li-Ion Battery Applications. *J. Phys. Energy* **2020**, *2* (3), 032006. https://doi.org/10.1088/2515-7655/ab9fe3.
(32) Li, L. Lattice Dynamics and Electronic Structures of $Ti_3C_2O_2$ and $Mo_2TiC_2O_2$ (MXenes): The Effect of Mo Substitution. *Computational Materials Science* **2016**, *124*, 8–14. https://doi.org/10.1016/j.commatsci.2016.07.008.
(33) Henkelman, G.; Uberuaga, B. P.; Jónsson, H. A Climbing Image Nudged Elastic Band Method for Finding Saddle Points and Minimum Energy Paths. *The Journal of Chemical Physics* **2000**, *113* (22), 9901–9904. https://doi.org/10.1063/1.1329672.
(34) Henkelman, G.; Jónsson, H. Improved Tangent Estimate in the Nudged Elastic Band Method for Finding Minimum Energy Paths and Saddle Points. *The Journal of Chemical Physics* **2000**, *113* (22), 9978–9985. https://doi.org/10.1063/1.1323224.
(35) He, R.; Wan, Y.; Zhao, P.; Guo, P.; Jiang, Z.; Zheng, J. First-Principles Investigation of Native Point Defects in Two-Dimensional Ti_3C_2. *Comput. Theor. Chem.* **2019**, *1150*, 26–39. https://doi.org/10.1016/j.comptc.2019.01.006.
(36) Sang, X.; Xie, Y.; Lin, M.-W.; Alhabeb, M.; Van Aken, K. L.; Gogotsi, Y.; Kent, P. R. C.; Xiao, K.; Unocic, R. R. Atomic Defects in Monolayer Titanium Carbide ($Ti_3C_2T_x$) MXene. *ACS Nano* **2016**, *10* (10), 9193–9200. https://doi.org/10.1021/acsnano.6b05240.
(37) Zhang, K.; Di, M.; Fu, L.; Deng, Y.; Du, Y.; Tang, N. Enhancing the Magnetism of 2D Carbide MXene Ti3C2Tx by H2 Annealing. *Carbon* **2020**, *157*, 90–96. https://doi.org/10.1016/j.carbon.2019.10.016.

(38) Gouveia, J. D.; Gomes, J. R. B. Structural and Energetic Properties of Vacancy Defects in MXene Surfaces. *Phys. Rev. Mater.* **2022**, *6* (2), 024004. https://doi.org/10.1103/PhysRevMaterials.6.024004.
(39) Wang, C.; Han, H.; Guo, Y. Stabilities and Electronic Properties of Vacancy-Doped Ti2CO2. *Computational Materials Science* **2019**, *159*, 127–135. https://doi.org/10.1016/j.commatsci.2018.12.007.
(40) Sang, X.; Xie, Y.; Yilmaz, D. E.; Lotfi, R.; Alhabeb, M.; Ostadhossein, A.; Anasori, B.; Sun, W.; Li, X.; Xiao, K.; Kent, P. R. C.; Van Duin, A. C. T.; Gogotsi, Y.; Unocic, R. R. In Situ Atomistic Insight into the Growth Mechanisms of Single Layer 2D Transition Metal Carbides. *Nat Commun* **2018**, *9* (1), 2266. https://doi.org/10.1038/s41467-018-04610-0.
(41) Wyatt, B. C.; Boebinger, M. G.; Hood, Z. D.; Adhikari, S.; Michałowski, P. P.; Nemani, S. K.; Muraleedharan, M. G.; Bedford, A.; Highland, W. J.; Kent, P. R. C.; Unocic, R. R.; Anasori, B. Alkali Cation Stabilization of Defects in 2D MXenes at Ambient and Elevated Temperatures. *Nat. Commun.* **2024**, *15* (1), 6353. https://doi.org/10.1038/s41467-024-50713-2.
(42) Persson, I.; Näslund, L.-Å.; Halim, J.; Barsoum, M. W.; Darakchieva, V.; Palisaitis, J.; Rosen, J.; Persson, P. O. Å. On the Organization and Thermal Behavior of Functional Groups on Ti_3C_2 MXene Surfaces in Vacuum. *2D Mater.* **2017**, *5* (1), 015002. https://doi.org/10.1088/2053-1583/aa89cd.
(43) Fang, H.; Thakur, A.; Zahmatkeshsaredorahi, A.; Fang, Z.; Rad, V.; Shamsabadi, A. A.; Pereyra, C.; Soroush, M.; Rappe, A. M.; Xu, X. G.; Anasori, B.; Fakhraai, Z. Stabilizing $Ti_3C_2T_x$ MXene Flakes in Air by Removing Confined Water. *Proc. Natl. Acad. Sci. U.S.A.* **2024**, *121* (28), e2400084121. https://doi.org/10.1073/pnas.2400084121.
